# Human brain organoid model of maternal immune activation identifies radial glia cells as selectively vulnerable

**DOI:** 10.1038/s41380-023-01997-1

**Published:** 2023-03-06

**Authors:** Kseniia Sarieva, Theresa Kagermeier, Shokoufeh Khakipoor, Ezgi Atay, Zeynep Yentür, Katharina Becker, Simone Mayer

**Affiliations:** 1 Hertie Institute for Clinical Brain Research, University of Tübingen, Tübingen, Germany; 2 International Max Planck Research School, Graduate Training Centre of Neuroscience, University of Tübingen, Tübingen, Germany; 3 Heidelberger Akademie der Wissenschaften, Heidelberg, Germany

**Keywords:** Neuroscience, Autism spectrum disorders, Stem cells, Molecular biology

## Abstract

Maternal immune activation (MIA) during critical windows of gestation is correlated with long-term neurodevelopmental deficits in the offspring, including increased risk for autism spectrum disorder (ASD) in humans. Interleukin 6 (IL-6) derived from the gestational parent is one of the major molecular mediators by which MIA alters the developing brain. In this study, we establish a human three-dimensional (3D) in vitro model of MIA by treating induced pluripotent stem cell-derived dorsal forebrain organoids with a constitutively active form of IL-6, Hyper-IL-6. We validate our model by showing that dorsal forebrain organoids express the molecular machinery necessary for responding to Hyper-IL-6 and activate STAT signaling upon Hyper-IL-6 treatment. RNA sequencing analysis reveals the upregulation of major histocompatibility complex class I (MHCI) genes in response to Hyper-IL-6 exposure, which have been implicated with ASD. We find a small increase in the proportion of radial glia cells after Hyper-IL-6 treatment through immunohistochemistry and single-cell RNA-sequencing. We further show that radial glia cells are the cell type with the highest number of differentially expressed genes, and Hyper-IL-6 treatment leads to the downregulation of genes related to protein translation in line with a mouse model of MIA. Additionally, we identify differentially expressed genes not found in mouse models of MIA, which might drive species-specific responses to MIA. Finally, we show abnormal cortical layering as a long-term consequence of Hyper-IL-6 treatment. In summary, we establish a human 3D model of MIA, which can be used to study the cellular and molecular mechanisms underlying the increased risk for developing disorders such as ASD.

## Introduction

The role of environmental factors in shaping prenatal brain development and contributing to neurodevelopmental disorders is being increasingly appreciated [1]. Epidemiological studies suggest that the health of the gestational parent (i.e., women in the experimental setup) correlates with fetal neurodevelopmental outcomes [2–4]. For example, infections lead to a higher probability to develop autism spectrum disorder (ASD) [5, 6] with an ASD hazard ratio of 2.98 (95% CI 1.24–7.15) due to viral infection in the first trimester and 1.42 (95% CI 1.08–1.87) for bacterial infection in the second trimester [5]. Considering the large number of COVID-19 cases, it will be important to consider long-term effects following prenatal exposure in the near future [7]. Common to different types of infection is the increase in cytokines and chemokines that, either via placental transfer or induction of placental inflammation and subsequent cytokine and chemokine release, leads to elevated concentrations of these immune mediators in the fetal compartment [7]. Elevated concentrations of the pro-inflammatory cytokine interleukin 6 (IL-6) during pregnancy have been correlated with changes in newborn brain anatomy, structural and functional connectivity, and altered cognitive function in infancy [8–10]. Taken together, there is mounting evidence for maternal immune activation (MIA) to affect neurodevelopmental trajectories and psychiatric disease risk in humans.

Diverse animal models have shown that MIA contributes to the behavioral deficits that recapitulate ASD symptoms in humans [11–15]. In mouse models, MIA is generally modeled by mimicking viral infection through a single injection of synthetic double-stranded RNA (dsRNA), polyinosinic:polycytidylic acid (poly(I:C)) at E12.5-E18 [16, 17]. Using gain and loss-of-function approaches in mouse models, several cytokines including IL-17α and IL-6 have been identified to be causal for behavioral deficits [11, 16, 17]. IL-17α mediates abnormal protein translation specifically in the neurons of male offspring resulting in behavioral deficits reminiscent of ASD [16]. IL-6 is necessary and sufficient to induce behavioral abnormalities in male offspring [11] and induces changes in the proliferation of radial glia cells (RGs) and differentiation of excitatory neurons in the neocortex [18]. Thus, mouse models have provided valuable insights into the molecular mechanisms of MIA implicating ASD-relevant changes in both neural progenitor cells and neurons [11, 16–19]. However, mouse models suffer from limitations including within-litter variability in behavioral responses [20] and a lack of translational potential to humans due to interspecies differences [21].

In vitro models now allow investigating MIA in a human cellular context. A recent study used human induced pluripotent stem cells (iPSCs)-derived neural precursor cells (NPCs) to show how interferon-γ (IFN-γ) activates the anti-viral response resulting in up-regulation of MHCI genes, targeting of MHCI proteins to the neuronal growth cone and, consequently, abnormal neurite outgrowth [22]. This effect is accompanied by ASD-relevant transcriptional abnormalities [22].

Recent advances in PSC-derived 3D brain organoid cultures provide new avenues to investigate the effects of environmental adversities on early brain development in a tissue context [21]. Organoids model key aspects of the cellular complexity of the developing human brain enabling direct observation of processes inaccessible *in utero* [23]. We propose that brain organoids are a powerful tool for studying human cell type-specific effects of molecular mediators of MIA.

**Fig. 1:**
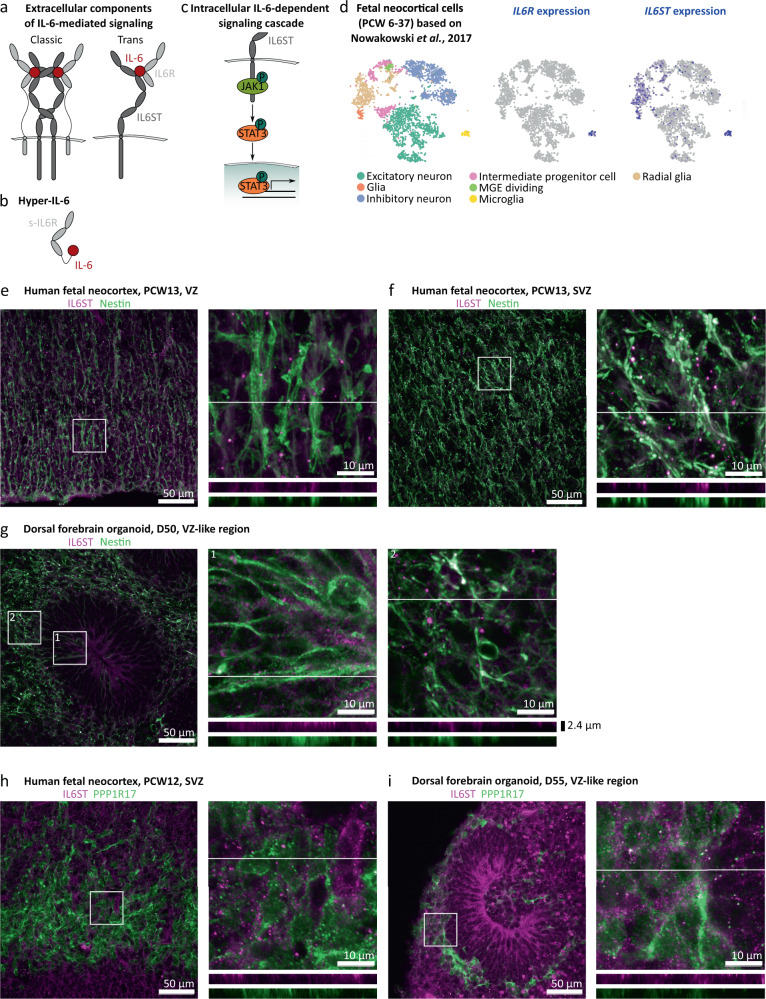
Characterization of the IL-6 signaling pathway in human midgestational neocortex and dorsal forebrain organoids. **a** IL-6 has two receptors, IL6R and IL6ST. When both of them are expressed in the target cell, the IL-6-dependent signaling cascade is called “classic” whereas when IL6R is provided extracellularly for example by a different cell in its soluble form, the cascade is called “trans”. **b** Hyper-IL-6 is a chimeric protein consisting of soluble IL6R (s-IL6R) and IL-6 connected to each other through a peptide linker. **c** Upon binding to IL6R and IL-6, IL6ST induces phosphorylation of JAK1, which in turn phosphorylates STAT3 at Y705. Then, p-Y705-STAT3 enters the nucleus and activates transcription. **d** In the developing human neocortex, *IL6R* is expressed exclusively in microglia whereas *IL6ST*expression can be detected in radial glia (RG) and to a lesser extent in intermediate progenitor cells (IPC), as well as in glial cells in a neocortical scRNAseq dataset (Nowakowski et al., *Science*, 2017). **e**, **f** IL6ST (magenta) immunofluorescence of human PCW13 neocortex, shows protein expression in the ventricular zone (VZ, **e**) and subventricular zone (SVZ, **f**) where it is associated with the marker of RGs Nestin (green). **g** IL6ST immunofluorescence is associated with Nestin in the VZ-like area of dorsal forebrain organoids (DFO) at day 50 of differentiation. **h** IL6ST (magenta) in human neocortex at PCW12 is weakly associated with PPP1R17 (green) which is used as a marker of IPCs. **i**, IL6ST immunofluorescence is weakly associated with PPP1R17 in DFOs at day 55 of differentiation. For **e**–**i**, the images represent maximum intensity projections of z-stacks sampled in 337 nm steps. The lines in the insets represent the positions from which orthogonal sections were sampled. Orthogonal sections for separate channels are shown underneath the respective inset images.

IL-6 acts on its target cells by binding to its specific receptor, interleukin 6 receptor (IL6R), which forms a complex with interleukin 6 cytokine family signal transducer (IL6ST), also known as gp130 (Fig. 1a) [24]. In classic signaling, IL6R and IL6ST are expressed by the same cell in *cis* [24]. However, IL6R can also be supplied in *trans* as a soluble form (s-IL6R) originating either from the alternative splicing of *IL6R* transcripts or from shedding from plasma membrane by metalloproteinases [24, 25]. Interestingly, the authors of a recent paper found that human iPSC-derived microglia constitutively release s-IL6R [26]. In the absence of microglia, *trans* signaling can be modelled by exposing target cells to the chimeric protein Hyper-IL-6 in which s-IL6R is covalently bound to IL-6 through a flexible peptide link (Fig. 1b) [27]. Upon binding of Hyper-IL-6 to IL6ST, intracellularly, IL6ST is autophosphorylated leading to the activation of the Janus kinase/signal transducer and activator of transcription (JAK/STAT) pathway [24] (Fig. 1c).

The IL6ST/JAK/STAT signaling pathway plays an important role in the developing neocortex, a brain region predominantly affected in many neurodevelopmental disorders including ASD [28]. IL6ST acts as an accessory receptor to other cytokines of the IL-6 family, including leukemia inhibitory factor (LIF), which contributes to maintaining the RG pool [29]. Indeed, the JAK/STAT pathway is activated in some RGs of the developing human neocortex at midgestation [30]. However, IL-6 itself is hardly expressed in the developing human and mouse brain [31, 32], and a rodent radioisotope tracing study suggests that IL-6 in the prenatal brain is primarily of maternal origin [33]. Therefore, maternal IL-6 signaling through JAK/STAT may be an important mediator of MIA in the developing human brain.

In this study, we developed a human 3D in vitro model of MIA using dorsal forebrain organoids (DFOs) [34] and activating IL-6 signaling to reveal the cellular and molecular responses to MIA during neocortical development. We used Hyper-IL-6 exposure for 5 to 10 days in DFOs starting at day 45 of differentiation to model MIA. This time point corresponds to the human neocortex at early midgestation. We treated cells for several days to account for the extended period of elevated cytokine levels upon exposure to infections. We validated our model by showing activation of JAK/STAT signaling upon Hyper-IL-6 exposure as well as upregulation of transcripts encoding proteins involved in the innate immune response. Immunohistochemical analysis of cell type composition revealed minor effects of Hyper-IL-6 treatment on the number of SOX2-positive ventricular RGs (vRGs). The long-term effects of Hyper-IL-6 treatment included an increased number of SATB2-positive upper-layer excitatory neurons (uExN) and laminar mislocalization of both CTIP2-positive deep-layer excitatory neurons (dExN) and SATB2-positive uExNs indicating a neuronal migration defect. Using single-cell RNA sequencing (scRNAseq) trajectory analysis, we found that exposure to Hyper-IL-6 led to a shift towards earlier progenitor cell types at the expense of neurons in the excitatory lineage. Hyper-IL-6 induced cell type-specific transcriptional changes, including upregulation of immune response-related genes and downregulation of genes involved in protein translation specifically in vRGs in agreement with previous animal and in vitro models of MIA. Moreover, our 3D model of MIA also revealed differentially expressed genes not reported in any other model, including *NR2F1*, which could have species-specific effects and may be especially relevant to link to human epidemiological findings. Therefore, our model is a needed extension to previous models of MIA and opens the door for further investigating causal relations underlying MIA-dependent neurodevelopmental trajectories in humans.

## Materials and Methods

See supplementary methods for more details.

### iPSC culture and DFO generation

Human iPSC lines BIONi010-C (Source: EBiSC) and HMGU1 (Source: Human Pluripotent Stem Cell Registry) were cultured in standard conditions. DFOs were generated as previously described with minor modifications [34].

### Human tissue samples

De-identified second-trimester tissue samples were collected with previous patient consent in strict observance of the legal and institutional ethical regulations. Protocols were approved by the Human Gamete, Embryo, and Stem Cell Research Committee (Institutional Review Board) at the University of California, San Francisco, and the institutional ethics committee at the University Hospital Tübingen. Tissues were fixed, cryopreserved, and cryosectioned at 16 µm as described in [35].

### Immunohistochemistry

Whole DFOs and primary tissues were processed for immunohistochemistry as previously described with minor modifications [36]. A complete list of antibodies used can be found in the supplement.

### Western Blotting

Organoid protein extraction was performed based on a previously published protocol [37]. Western Blotting detection was performed with the Li-COR Odyssey system according to the manufacturer’s instructions.

### RNA sequencing

mRNA sequencing was performed by Novogene and samples were prepared according to the provider’s instructions.

### Single-cell dissociation and multiplexing for scRNAseq

Individual DFOs were dissociated following a published protocol [38] with minor modifications. After dead cell removal (Miltenyi, Cat. no. 130-090-101), cells were subjected to multiplexing using 3′ CellPlex Kit Set A (10x Genomics, Cat. no. PN-1000261) following the manufacturer’s instructions. Equal proportions were loaded onto a Chromium Single Cell 3′ Chip (10x Genomics, Cat. no. PN-120236) and processed through the Chromium controller to generate single-cell gel beads in emulsion. scRNAseq libraries were prepared with the Chromium Single Cell 3′ Library & Gel Bead Kit v.3 (10x Genomics, Cat. no. PN-1000121). Libraries from different samples were pooled and sequenced on a NovaSeq6000 instrument (Illumina).

### scRNAseq analysis

scRNAseq analysis was performed using a range of publicly available tools as detailed in the Supplementary Methods.

### Statistics

Statistical tests used are indicated in each figure legend. Cell line of origin and batch of organoid differentiation were included as a covariate in the models unless stated otherwise.

## Results

### RG in the human neocortex at midgestation express the molecular machinery to respond to IL-6

To determine cell types susceptible to IL-6 in the developing human neocortex at midgestation, a peak period of neurogenesis, we first analyzed the expression of the IL-6 receptors, *IL6R* and *IL6ST*, in a scRNAseq dataset [31]. We found that *IL6R* was expressed exclusively in microglia (Fig. 1d), whereas *IL6ST* was predominantly present in both microglia and RGs (Fig. 1d). We confirmed IL6ST expression in the ventricular (VZ) and subventricular (SVZ) zones in situ (Fig. 1e,f) and found association with the RG marker Nestin (Fig. 1e,f, Supplementary Fig. 2a) and to a lesser extent with intermediate progenitor cell (IPC) marker PPP1R17 (Fig. 1h, Supplementary Fig. 1b). As previously reported [30], we found that p-Y705-STAT3 co-localized with SOX2, a marker of RGs, in a subset of cells in both VZ and SVZ (Supplementary Fig. 1c). We therefore concluded that RGs at midgestation might be susceptible to IL-6 through *trans* signaling (Fig. 1a) with s-IL6R supplied by microglia.

Similarly, human DFOs generated using a published protocol [34] at day 50 of differentiation express IL6ST in the Nestin-positive RGs localized to the VZ-like and SVZ-like areas (Fig. 1g) and, to a lesser extent, in the PPP1R17-positive IPCs (Fig. 1i). At this stage, organoids consist mainly of SOX2-positive RGs organized into rosettes, TBR2-positive IPCs, and CTIP2-positive dExNs (Supplementary Fig. 1d). This cellular arrangement corresponds to the human neocortex at early midgestation (Supplementary Fig. 1e) [31], but lacks cells of non-neuroectodermal identity (i.e., microglia, blood vessels) and has an under-representation of the cells of non-dorsal forebrain origin that migrate into the neocortex such as interneurons [39].

### Establishment and characterization of IL-6 treated DFOs as a model of MIA

**Fig. 2:**
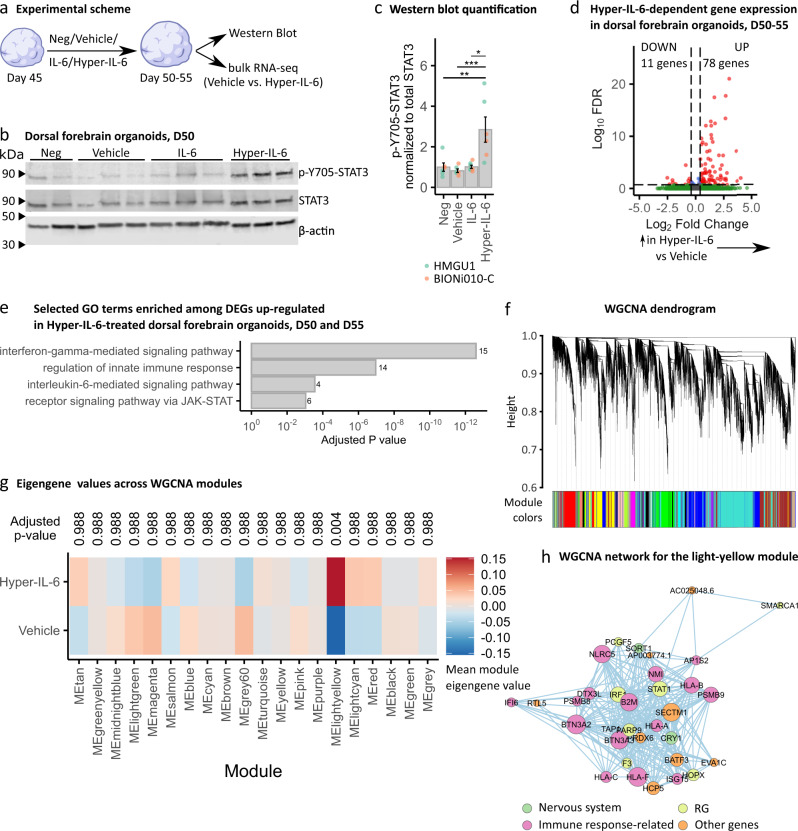
Hyper-IL-6 treatment leads to the activation of JAK/STAT intracellular cascade and results in transcriptional changes in dorsal forebrain organoids. **a** Scheme of the cytokine treatment in DFOs. Neg, negative control; Vehicle, vehicle control (0.1% BSA in PBS); IL-6, 8.8 ng/ml in 0.1% BSA in PBS; Hyper-IL-6, 25 ng/ml in 0.1% BSA in PBS. **b** Representative Western Blot of p-Y705-STAT3 (top), STAT3 (middle) and β-actin (bottom) in individual DFOs at day 50 of differentiation after 5 days of treatment. **c** Quantification of signal intensity of p-Y705-STAT3 relative to total STAT3 from **b**. Each dot represents an individual organoid sample. Color represents cell line of origin, HMGU1 (green), BIONi010-C (orange). Neg (*n* = 6), Vehicle (*n* = 6), IL-6 (*n* = 7), and Hyper-IL-6 (*n* = 6) DFOs from one batch per iPSC line. Bars represent mean, error bars represent ± SEM. Comparisons were analyzed using Aligned Rank Transform (ART) ANOVA: *p-value < 0.05, **p-value < 0.01; ***p-value < 0.001. **d** Volcano plot of Hyper-IL-6-dependent gene expression in DFOs at days 50 and 55 of differentiation treated for 5 to 10 days. Red dots indicate statistical significance (FDR < 0.2, absolute log2 Fold Change >0.4). Positive log2 Fold Change indicates higher gene expression in Hyper-IL-6-treated relative to Vehicle-treated DFOs. Data from *n* = 12 organoids per condition (two cell lines, day 50 and 55 analyzed together). **e** GO overrepresentation analysis (ORA) of differentially upregulated genes (FDR < 0.2, log2 Fold Change >0.4) between Hyper-IL-6- and Vehicle-treated DFOs. The x-axis displays the adjusted *p*-value. Numbers next to the bars represent the number of the differentially expressed genes (DEG) belonging to the GO term. Data from *n* = 12 organoids per condition (two cell lines, day 50 and 55 analyzed together). **f** Dendrogram for WGCNA obtained by clustering the dissimilarity based on consensus Topological Overlap. Each color represents a module, which contains a group of highly connected genes. A total of 20 modules were identified. **g** Heatmap representing mean module eigengene value by treatment condition based on WGCNA. Comparisons were analyzed using *t*-test, Bonferroni adjusted *p*-value. **h** WGCNA network for the light-yellow module. Color of the dots represents gene group: nervous system-enriched, green; radial glia-enriched, yellow; immune-response-related, pink; other genes, orange.

To investigate the effect of IL-6 exposure on human neocortical development, we differentiated human DFOs from two male iPSCs lines since pronounced effects are expected to be found in males based on animal studies [16] and ASD prevalence in humans [36]. We exposed DFOs to 8.8 ng/ml (422.6 nM) IL-6 and equimolar concentration of Hyper-IL-6 (25 ng/ml, 422.6 nM) for 5 to 10 days starting at day 45 of differentiation to model the period of early midgestation. This concentration is comparable to the serum concentration of IL-6 upon septic infection [40]. The prolonged treatment scheme was chosen because IL-6 serum concentrations may be elevated beyond acute inflammatory response [41]. 0.1% BSA diluted in PBS was used as a Vehicle solution for both IL-6 and Hyper-IL-6 to enhance the stability of recombinant proteins (see Fig. 2a for experimental design). To assess the activation of the signaling cascade downstream of IL-6/Hyper-IL-6 treatment, we analyzed phosphorylation of STAT3 at Y-705 relative to total STAT3 using Western Blot (Fig. 2b). We found that treatment with Hyper-IL-6, but not IL-6, resulted in an increased p-Y705-STAT3 (Fig. 2c). We also showed that p-Y705-STAT3 co-localized with SOX2 in the VZ-like areas after Hyper-IL-6 treatment (Supplementary Fig. 2a). We, therefore, concluded that, as predicted by gene expression data (Fig. 1), DFOs were capable of responding to Hyper-IL-6 treatment through *trans-*signaling. Therefore, we reasoned that Hyper-IL-6 treatment could be used to model MIA in DFOs.

STAT3 is a transcriptional activator with cell type-specific targets in human neurodevelopment [30, 42, 43]. Moreover, MIA has been shown to induce transcriptional changes in rodent models [16, 44]. Therefore, we next investigated the transcriptional changes associated with the exposure of DFOs to Hyper-IL-6 by performing RNAseq on days 50 and 55 of differentiation (Supplementary Fig. 2b,c, Supplementary Table 1). To identify gene expression changes driven by Hyper-IL-6, we performed differential gene expression (DGE) analysis. DGE analysis was performed with relatively permissive significance thresholds (FDR ≤ 0.2, absolute log2 Fold Change ≥ 0.4) to correct for relatively low number of samples as reported in a similar study [45]. First, we analyzed DGE separately at two time points. We identified 123 and 3104 DEGs on day 50 and day 55, respectively (Supplementary Fig. 3d, Supplementary Table 2,3; FDR ≤ 0.2, absolute log2 Fold Change ≥ 0.4; three DFOs from two iPSC lines per time point) which belonged to different functional groups as shown by gene set overrepresentation (ORA) and enrichment analyses (GSEA) (Supplementary Fig. 3e, Supplementary Table 4,5). Second, we performed DGE analysis without considering dosing length as a covariate. We identified 89 differentially expressed genes (DEGs) (Fig. 2d; FDR ≤ 0.2, absolute log2 Fold Change ≥0.4; three DFOs from two iPSC lines in two differentiation experiments) of which only 11 showed a downregulation (Supplementary Table 6). The upregulated DEGs were associated with innate immune response (e.g., MHCI members and *B2M*) as well as with response to IL-6 and signaling through the JAK/STAT pathway based on ORA (Fig. 2e and Supplementary Table 7). Interestingly, MHCI components and its accessory B2M protein have been reported to be also deregulated upon IFN-γ treatment in human iPSC-derived NPCs [22].

Since gene expression has a correlative structure it can be analyzed as gene co-expression modules [38] to enable functional interpretation of the DEGs [46]. We, therefore, employed weighted gene co-expression network analysis (WGCNA) (Fig. 2f). WGCNA revealed 20 modules of genes with correlated expression. Only the light-yellow module was associated with Hyper-IL-6 treatment (Fig. 2g). A network connection representation of the genes constituting the light-yellow module showed an overlapping expression of RG-enriched genes [30] with genes involved in immune response (Fig. 2h, Supplementary Fig. 2f and Supplementary Table 8). Therefore, RGs are likely the most susceptible cell type to Hyper-IL-6 exposure, in line with the expression pattern of IL6ST (Fig. 1).

### Mild cytoarchitectural changes are observed in Hyper-IL6-treated DFOs

**Fig. 3:**
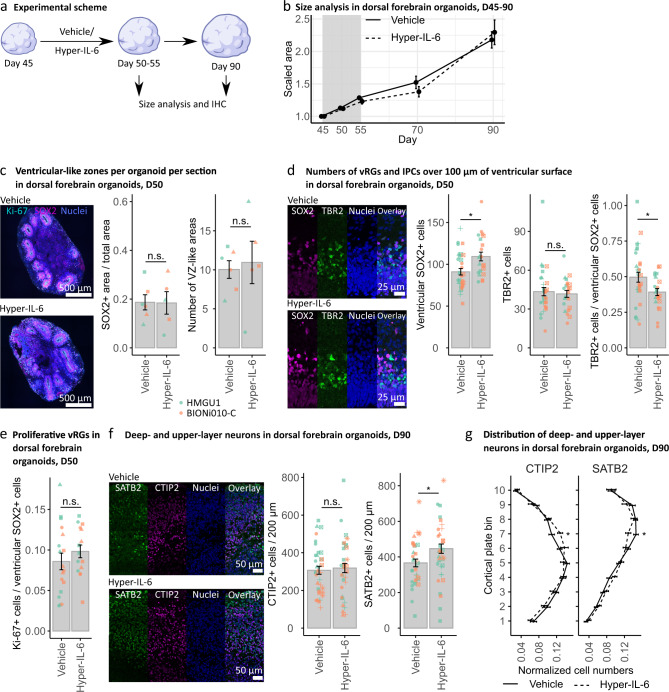
Hyper-IL-6 treatment results in subtle changes in cell type composition in dorsal forebrain organoids. **a** Experimental timeline and sample harvesting scheme. **b** Hyper-IL-6 treatment does not lead to changes in size of the DFOs during the treatment (days 45–55, grey area) and at least up to day 90 of differentiation. Vehicle (*n* = 5–32) and Hyper-IL-6 (*n* = 5–32) DFOs from one batch per iPSC line, two iPSC lines. **c** Hyper-IL-6 treatment does not lead to changes in the proportion of the SOX2-positive areas and the number of the VZ-like regions in the DFOs. Ki-67 (cyan), SOX2 (magenta), nuclei stained by DRAQ5 (blue). Four sections of each DFO were analyzed. Each dot represents the mean value of four sections in a single organoid. Color represents cell line of origin, HMGU1 (green), BIONi010-C (orange). Vehicle (*n* = 6) and Hyper-IL-6 (*n* = 5) DFOs from one batch per iPSC line. **d** Hyper-IL-6 treatment leads to an increase in SOX2-positive ventricular RGs (vRG). Hyper-IL-6 treatment does not change the number of TBR2-positive IPCs. Hyper-IL-6 treatment leads to decreased proportion of TBR2-positive IPCs over SOX2-positive vRGs. Each dot represents individual VZ/SVZ-like region, 2–6 regions imaged per organoid. Color represents cell line of origin, HMGU1 (green), BIONi010-C (orange). Shape corresponds to individual DFO. Vehicle (*n* = 8) and Hyper-IL-6 (*n* = 6) DFOs from one batch per iPSC line. **e** Hyper-IL-6 treatment does not lead to changes in proportion of Ki-67-positive proliferative cells over SOX2-positive vRGs. Each dot represents individual VZ-like region, 2–3 regions imaged. Color represents cell line of origin, HMGU1 (green), BIONi010-C (orange). Shape corresponds to individual DFO. Vehicle (*n* = 6) and Hyper-IL-6 (*n* = 5) DFOs from one batch per iPSC line. **f** Hyper-IL-6 treatment does not change the number of CTIP2-positive deep-layer excitatory neurons (dExN) at day 90 of differentiation while increasing the number of SATB2-positive upper-layer excitatory neurons (uExN). Each dot represents an individual region-of-interest representing a putative cortical plate (CP)-like region, 2-3 regions per DFO imaged. Color represents cell line of origin, HMGU1 (green), BIONi010-C (orange). Shape corresponds to individual DFO. Vehicle (n = 12) and Hyper-IL-6 (*n* = 14) DFOs from three batches. **g** Hyper-IL-6 treatment leads to incorrect laminar positioning of both CTIP2-positive dExNs and SATB2-positive uExNs at day 90 of differentiation. The cortical structure from the surface of the organoid until the end of dense CTIP2-positive area was evenly divided into bins 1–10. Shown are curves representing the normalized abundance within each bin, calculated as [#CTIP2/SATB2+ cells in a bin/#total CTIP2/SATB2+ cells]. Same samples as in **f** are shown. In all panels, bars (for **c**–**f**) and points (for **b** and **g**) represent mean, error bars represent ± SEM. For panels **b**–**f**, comparisons were analyzed using Aligned Rank Transform (ART) ANOVA: n.s., non-significant *p*-value >0.05, *p-value < 0.05. For panel **g**, comparisons were analyzed using *t*-test: *p-value < 0.05.

Our findings suggested that Hyper-IL-6 treatment was mostly targeting RGs within DFOs. Previous studies have indicated that MIA in mice alters the proportions of proliferative versus neurogenic cell divisions at E12.5 culminating in an overproduction of dExNs later in development [18]. To evaluate potential disturbances in the cell fate decisions of RGs upon exposure to Hyper-IL-6, we performed a series of immunohistochemical analyses to assess organoid morphology and cell type composition changes (Fig. 3a). First, we followed the growth rate of the DFOs upon Hyper-IL-6 treatment on days 45–55 and until day 90 of differentiation. We observed no differences either in the organoid size (Fig. 3b) or area, perimeter, or number of SOX2-positive VZ-like regions within individual organoids at days 50 and 55 of differentiation (Fig. 3c and Supplementary Fig. 2a, b). However, the number of SOX2-positive cells within individual VZ-like regions was higher in Hyper-IL-6-exposed organoids (Fig. 3d). While the number of TBR2-positive IPCs was constant, the proportion of IPCs to SOX2-positive RGs was decreased (Fig. 3d). The proliferation rate of vRGs, measured as the proportion of Ki-67-positive cells to SOX2-positive cell count, was not altered (Fig. 3e and Supplementary Fig. 3c). We also observed no differences in the count of CTIP2-positive dExNs and the total Tuj1-positive neuron-enriched area upon Hyper-IL-6 exposure at day 50 of differentiation (Supplementary Fig. 3d). Together, these data suggest minor changes in the counts of SOX2-positive RGs, which do not result in an overproduction of IPCs or neurons in a short time frame. We also investigated the long-term effect of the Hyper-IL-6 treatment by analyzing the number of CTIP2-positive dExNs and SATB2-positive uExNs at day 90 of differentiation (Fig. 3f). We found no significant changes in CTIP2-positive cell counts whereas the number of SATB2-positive cells was increased upon Hyper-IL-6 exposure (Fig. 3f). MIA in mouse models leads to abnormalities not only in the number of neurons but also in the cortical cytoarchitecture [17, 18]. Therefore, we characterized two aspects of cortical cytoarchitecture. First, we analyzed the proportion of double CTIP2-, SATB2-positive cells to the total number of either CTIP2- or SATB2-positive cells to assess fate misspecification upon Hyper-IL-6 treatment. We did not find any difference between the experimental conditions (Supplementary Fig. 3e). Next, we analyzed the distribution of both CTIP2- and SATB2-positive cells along the putative cortical plate (CP) to assess cortical layering (Fig. 3g and Supplementary Fig. 3f). CTIP2-positive dExNs were overrepresented in the upper bins of putative CP while SATB2-positive uExNs were overrepresented in the lower bins of putative CP upon Hyper-IL-6 treatment (Fig. 3g). These mild laminar positioning abnormalities indicate a neuronal migration or maturation defect. Coherent with the absence of clinical observations of the gross anatomical malformations in neocortical development upon MIA, we show that Hyper-IL-6 treatment of DFOs leads to mild cytoarchitectural changes after 6-7 weeks in culture.

### Lineage trajectory analysis in scRNAseq data shows shift towards earlier pseudoage

**Fig. 4:**
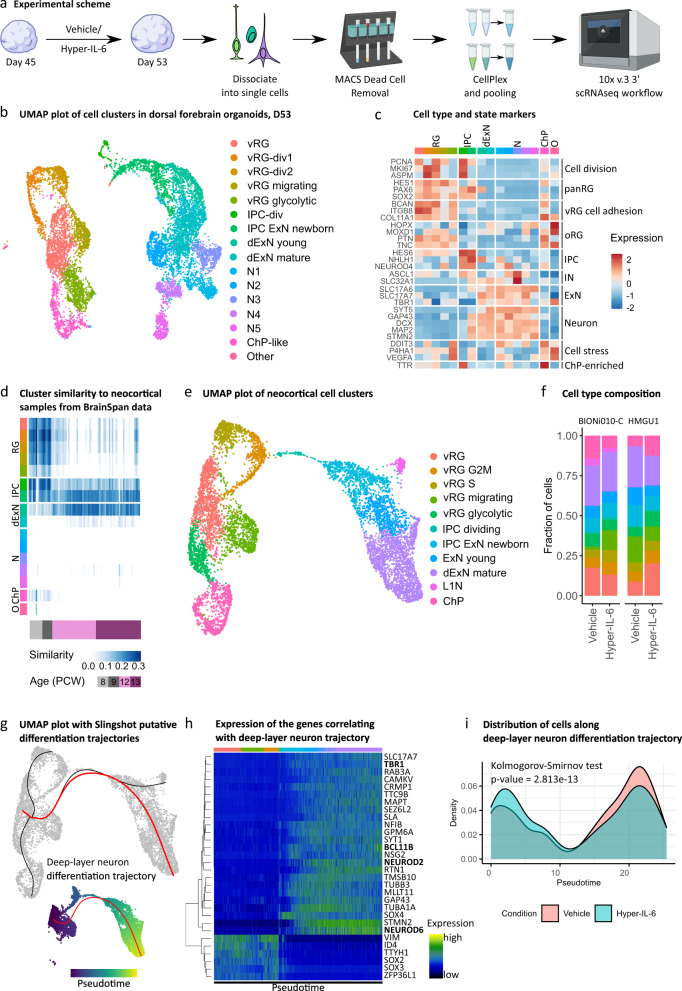
Single-cell RNA sequencing of the dorsal forebrain organoids at day 53 of differentiation upon Hyper-IL-6 and Vehicle treatment. **a** Schematic overview of the experimental design, including dissociation of DFOs, dead cell removal and sample multiplexing by CellPlex. **b** UMAP with 16 clusters with cell types labeled by color. Data from *n* = 2 organoids per condition. vRG, ventricular radial glia; vRG-div, dividing ventricular radial glia; IPC, intermediate progenitor cell; dExN, deep-layer excitatory neuron; N, neuron; ChP, choroid plexus. **c** Heatmap of gene expression level for selected known markers of neural cell types across clusters. Colors in the upper bar represent cell types from **b**. oRG, outer radial glia; IN, inhibitory neuron. **d** Heatmap of similarity metric of VoxHunt algorithm comparing organoid clusters with human neocortical RNAseq data from BrainSpan using brain regional markers obtained from Mouse Brain Atlas at E13. Colors in the left bar represent cell types from **b**. Data from *n* = 4 organoids. **e** UMAP with 11 clusters resulting from subclustering of cells with dorsal and medial forebrain regional identity. **f** Bar plots of the proportions of cell types by organoid in two cell lines. **g** Putative differentiation trajectories (generated by Slingshot) overlaid over UMAP, dExN differentiation trajectory in red. **h**, Heatmap of gene expression of genes correlating with progression through pseudotime over dExN differentiation trajectory as assessed by random forest classifier. Colors in the bar correspond to cell types from **e**. Hierarchical clustering of the genes shown on the left. Data from *n* = 4 organoids. **i** Density plot of cells over dExN differentiation trajectory by experimental condition. Kolmogorov-Smirnov test. Data from *n* = 2 organoids per condition.

Taken together, our transcriptomic and immunohistochemical analysis indicated that there might be cell type-specific responses to Hyper-IL-6 treatment with strongest effects expected in RGs. To investigate whether Hyper-IL-6 exposure results in cell type-specific molecular changes, we treated DFOs using our established paradigm and profiled individual organoids using scRNAseq at day 53 of differentiation (Fig. 4a). We profiled two organoids per experimental condition originating from two male iPSCs lines. After quality and doublet filtering (quality metrics provided in Supplementary Table 9 and Supplementary Fig. 4a), we analyzed the transcriptomes of 11 162 cells, recovering an average of 3200 unique transcripts per cell. Using Louvain clustering, we identified 16 transcriptionally distinct clusters (Fig. 4b). A combination of DGE analysis, analysis of expression of common cell type markers and gene module expression analysis allowed us to characterize all clusters (Supplementary Table 10,11). Using this approach, we identified multiple types of progenitor cells and neurons as well as cell states, including proliferative and metabolic states (Fig. 4c and Supplementary Fig. 4b). Importantly, cell type classifications were in broad agreement with previous scRNAseq data from DFOs and primary neocortex [34, 47, 48] (Supplementary Fig. 4c). Next, we assessed the neocortical identity of different clusters using VoxHunt [49]. First, we used the E13 Allen Developing Mouse Brain Atlas dataset as a reference for the expression of regional identity markers [50]. These markers were further employed to correlate transcriptomes of our cell clusters with human neocortical transcriptomes from BrainSpan [51]. This analysis revealed a subset of neuronal clusters whose regional marker expression did not correlate with neocortical markers but corresponded to ventral forebrain or more caudal brain regions (Fig. 4d and Supplementary Fig. 4d). Cells originating from these non-neocortical neuronal clusters were enriched in organoids generated from one iPSC line (Supplementary Fig. 4e) and, therefore, omitted from further analysis resulting in 8 130 cells with dorsal and medial forebrain identity constituting the final dataset. We reclustered these cells for downstream analysis and obtained 11 clusters (Fig. 4e and Supplementary Table 12). Analysis of cell type proportions within individual organoids did not reveal major shifts in cellular composition upon Hyper-IL-6 treatment (Fig. 4f and Supplementary Fig. 4f). However, the reconstruction of the dExN lineage trajectory showed a shift towards earlier pseudoage in Hyper-IL-6-treated organoids (Fig. 4g–i) in line with immunohistochemical characterization (Fig. 3).

### Differential gene expression analysis suggests disturbed protein translation in vRGs

**Fig. 5:**
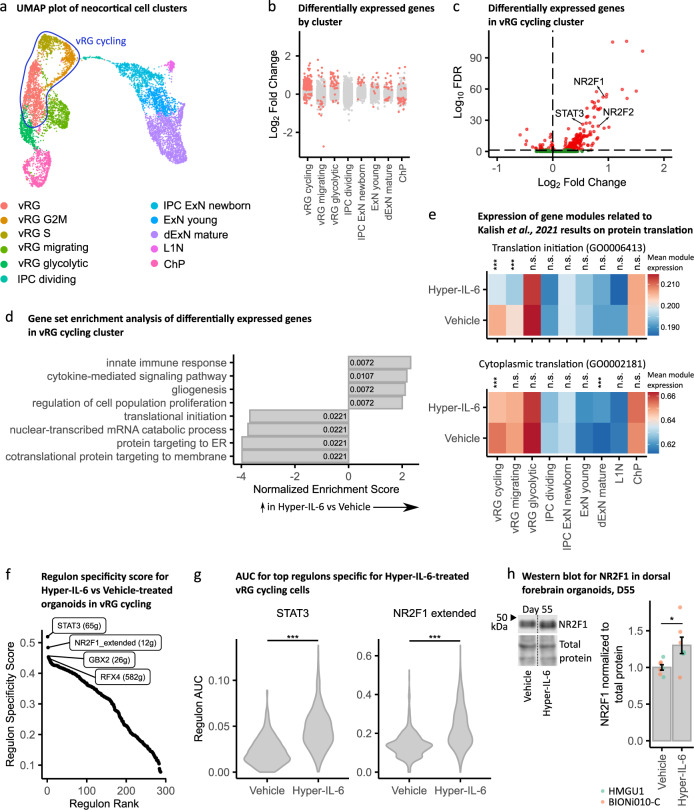
Single-cell DGE and transcriptional networks change upon Hyper-IL-6 treatment. **a** vRG, vRG S and vRG G2M clusters unified into cycling vRG metacluster in the UMAP with clusters with cell types resembling forebrain regional identity from Fig. 4e. **b** Strip plot displaying DEGs between Hyper-IL-6 and Vehicle-treated organoids in red (FDR < 0.05). The x axis displays cell clusters from **a**. L1N is missing because these cells were not present in all samples. **c** Volcano plot of Hyper-IL-6-dependent gene expression in cycling vRG metacluster. Red dots indicate statistical significance (FDR < 0.05). Positive log2 Fold Change indicates higher gene expression in Hyper-IL-6-treated relative to Vehicle-treated cells. **d** Gene set enrichment analysis (GSEA) of DEGs (FDR < 0.05) between Hyper-IL-6 and Vehicle-treated cycling vRGs from **c**. The x-axis displays normalized enrichment score. Numbers inside the bars represent adjusted *p*-values for differential enrichment. **e**, Heatmap representing mean module expression assessed by UCell by treatment condition across cell clusters from **a**. Comparisons were analyzed using *t*-test, Bonferroni adjusted *p*-values: ***p-value < 0.001. **f** Rank for regulons in Hyper-IL-6-treated cycling vRGs based on regulon specificity score (RSS) found by SCENIC. **g**, Regulon area under the curve (AUC) for STAT3 and NR2F1-driven regulons in cycling vRGs between conditions. Comparisons were analyzed using *t*-test: ***p-value < 0.001. **h** Representative Western Blot image and quantification measuring NR2F1 (top) normalized to Total Protein Stain (bottom) in dorsal forebrain organoids at day 55 of differentiation. In the plot, each dot represents an individual organoid. Color represents cell line of origin, HMGU1 (green), BIONi010-C (orange). Vehicle (*n* = 7) and Hyper-IL-6 (*n* = 7) DFOs from one batch per iPSC line. Bars represent mean, error bars represent ± SEM. Comparisons were analyzed using Aligned Rank Transform (ART) ANOVA: *p-value < 0.05.

We aimed to characterize cell type-specific transcriptional alterations upon Hyper-IL-6 treatment by performing DGE analysis. In order to focus on cell types rather than cell states, we first unified clusters of vRGs belonging to different stages of the cell cycle into a “cycling vRG” metacluster (Fig. 5a). Although all clusters had DEGs, we identified a higher number of DEGs in the cycling vRGs compared to all other cell clusters (Fig. 5b,c and Supplementary Table 13). Among the upregulated DEGs in the cycling vRGs, we identified transcription factors (TFs) *STAT3*, *NR2F1*, and *NR2F2*, among others, based on comparison with the human TFs list [52] (Fig. 5c). Gene set enrichment analysis (GSEA) of DEGs suggested innate immune response-related genes as well as genes involved in the regulation of cell proliferation and gliogenesis being upregulated (Fig. 3d and Supplementary Table 14). This result agrees with previous reports showing that JAK/STAT pathway activation is pro-proliferative and -gliogenic in NPCs [42]. Among the gene ontology (GO) terms enriched in downregulated DEGs of cycling vRGs, we found translation initiation and protein targeting to ER (Fig. 3d). We also aimed to link differential gene expression in the study by Kalish and colleagues to our data. We, therefore, identified gene lists behind the GO terms that were significant in GO enrichment analysis in the mouse model of MIA [16] and performed module expression of these genes in our dataset. We found significant downregulation of translation initiation and cytoplasmic translation in cycling vRG while cytoplasmic translation was upregulated in dExNs (Fig. 5e). Downregulation of protein translation-related genes in cycling vRG is in agreement with the widespread disruption of protein translation in mouse embryos upon MIA (Supplementary Fig. 5a,b) [16, 44]. Downregulation of protein translation in neurons was suggested to be causal for mediating behavioral abnormalities [16]. Here, we provide evidence that abnormal protein translation manifests already on the level of RGs preceding potential abnormalities in neurons.

To analyze the relevance of the DEGs for ASD, we performed two types of analysis. First, gene module expression analysis of the ASD-relevant gene groups reported by Satterstrom and colleagues in our scRNAseq dataset revealed significant deregulation of gene groups “Cytoskeleton” and “Neuronal communication” specifically in RGs (Supplementary Fig. 5c) [53]. However, SFARI genes of categories 1–4 by the old classification and categories 1-2 by the new classification were not differentially enriched among DEGs in our single-cell clusters (Supplementary Fig. 5d).

We further performed SCENIC analysis to better characterize gene networks deregulated by Hyper-IL-6 treatment specifically in vRG cycling cells [54, 55]. The highest specificity scores were obtained by STAT3 and NR2F1-driven regulons (Fig. 5f and Supplementary Table 15). The activity of these regulons was significantly higher in Hyper-IL-6-treated cells (Fig. 5g). While the increased activity of STAT3-mediated gene expression further validates our model (Supplementary Fig. 5e and Supplementary Table 16) in line with RNAseq and Western Blot data, NR2F1 has not been reported as differentially expressed in rodent models of MIA [16, 44]. ORA of the genes in NR2F1 regulon revealed genes involved in synapse organization and cell migration (Supplementary Fig. 5f, g, Supplementary Table 16). We confirmed the upregulation of NR2F1 on the protein level in Hyper-IL-6-treated organoids at day 55 of differentiation in Western Blot (Fig. 5h) and showed that NR2F1 is expressed across cell types in the DFOs at day 50 of differentiation by immunohistochemistry (Supplementary Fig. 5h). Additionally, RNAseq data also showed an upregulation of *NR2F1* in Hyper-IL-6-treated organoids at day 55 of differentiation (log2 Fold Change = 0.71, adjusted FDR = 0.11).

## Discussion

**Fig. 6:**
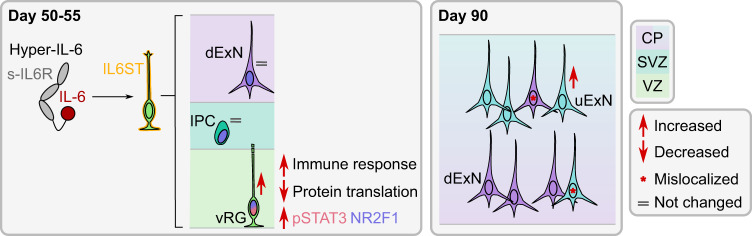
Summary of cellular and molecular effects observed in DFOs upon Hyper-IL-6 treatment. In the DFOs, RG cells express IL6ST and are, therefore, capable of responding to Hyper-IL-6 treatment. IL6ST activation results in phosphorylation of Y705-STAT3 and elevated number of vRGs without an effect on the IPCs and dExNs numbers at days 50–55 of organoid differentiation. Downstream, immune response-related gene expression is activated while protein translation gene expression is downregulated in vRGs. Hyper-IL-6 treatment results in cell type-independent upregulation of *NR2F1*. The protracted effects of Hyper-IL-6 treatment (day 90 of organoid differentiation) include increased number of uExNs and laminar mislocalization of both dExNs and uExNs.

By establishing a human 3D model of the cytokine-mediated effects of MIA on human neocortical development (Fig. 6), we close the gap between human epidemiological studies and mechanistic studies in animal models.

### IL-6 as a molecular mediator of MIA

Epidemiological studies, as well as animal and 2D in vitro models of MIA, have revealed possible molecular mediators of MIA, including IL-6, IL-17α, TNF-α, and IFN-γ, that drive the adverse neurodevelopmental outcomes [11, 16, 17, 22, 56] with IL-6 playing a key role [11, 18]. We, therefore, chose to model MIA by activating IL-6 signaling. To show the validity of this approach, we confirmed that the molecular machinery required for responding to IL-6 is expressed in the developing human neocortex as well as DFOs. Since only microglia express *IL6R* in the developing human brain [31], we hypothesized that neuroectodermal cells can only be activated by IL-6, if IL6R is provided in *trans* from microglia. We model *trans*-signaling in DFOs devoid of microglia by applying Hyper-IL-6, a chimeric protein consisting of the soluble IL6R and IL-6 connected by an oligopeptide linker [57]. We observed that only Hyper-IL-6, not IL-6 itself, activates JAK/STAT signaling validating our hypothesis (Fig. 2). Therefore, we chose to model MIA with Hyper-IL-6 in DFOs devoid of microglia. We suggest that a similar *trans-*signaling mechanism may also link MIA-induced IL-17α elevations to neurodevelopmental outcomes [16, 17], since transcriptomics data suggests that *IL17RA* is expressed predominantly in microglia [31, 32]. In the future, our model can be expanded by incorporating microglia in DFOs [58–60].

### Cellular effects of MIA on neocortical development: modulation of neurogenesis

Infections during the first and early second trimester pose the highest risk for developing neurodevelopmental deficits [5]. We, therefore, focus on an early time point in organoid differentiation, that models early midgestation [31]. Infections usually last several days, prompting us to treat organoids for 5 to 10 days. Immunohistochemical and scRNAseq analysis showed increased numbers of vRGs, while not significantly affecting IPCs and neurons within days of treatment (Figs. 3, 4). Similarly, in a mouse model of MIA, higher numbers and proliferative capacity of vRGs was observed [18]. In the mouse MIA also led to an overproduction of dExNs by E18.5 [18]. When we quantified neuronal populations on Day 90, 35 days after the end of Hyper-IL-6 treatment, we found that while the number of CTIP2-positive dExNs was unchanged, the number of SATB2-positive uExNs was significantly higher in Hyper-IL-6-treated organoids (Fig. 3). Moreover, similar to the mouse model of MIA [18], the CTIP2-positive dExNs were mislocalized in the upper layers of putative CP. Additionally, and coherent with another study [17], the SATB2-positive uExNs were mislocalized to deeper layers of the putative CP. Together, these observations indicate a migration or maturation defect induced by Hyper-IL-6 treatment. The discrepancies between mouse and organoid models of MIA may be due to species-specific differences in the cellular responses to MIA, differences between the in vivo and in vitro setting, or differences in the extent of MIA induced by poly(I:C) and Hyper-IL-6 treatment. We model the effect of MIA in the early phase of human neocortical neurogenesis. We expect that our model will be used to reveal how MIA affects later neurodevelopmental processes in a human context, focusing for example on outer or basal RGs, which are abundant in the human but almost absent from the mouse neocortex [61–63].

### Molecular effects of MIA on neocortical development: changes in gene expression associated with immune response, protein translation, and NR2F1 signaling

In order to determine which molecular changes were induced in our MIA model and how they compared mechanistically to previous findings in other models, we performed transcriptomic analysis. In RNAseq data, the top upregulated genes were at the interface of innate and adaptive immune responses: MHCI genes and *B2M*, which encodes the ancillary protein necessary for the MHCI assembly [64]. Analogously, Warre-Cornish and colleagues also found expression of MHCI proteins in iPSC-derived neural precursor cells treated with IFN-γ [22]. This resulted in abnormal neurite outgrowth, which was rescued by a *B2M* knockdown in differentiating neurons [22]. The authors note that similarly, iPSC-derived neurons from patients with ASD show neurite outgrowth deficits [65–67]. In our study, WGCNA analysis showed co-expression of MHCI genes with RG-enriched genes (Fig. 2) suggesting a similar mechanism of IL-6 action as found for IFN-γ-treated NPCs. Upregulation of MHCI proteins was also reported in a mouse model of MIA [68] and *HLA-A* alleles are differentially associated with ASD in humans [69]. Therefore, our data further strengthens the link between the cellular effects of MIA and neurodevelopmental processes altered in ASD [22, 68].

Since our WGCNA analysis indicated that RGs might be specifically affected by Hyper-IL-6 treatment (Fig. 2), we performed scRNAseq. Indeed, we found the highest number of differentially expressed genes in the vRGs (Fig. 5). Among the downregulated genes were many involved in protein translation, consistent with the results obtained from diverse cell types including RGs in a mouse model of MIA [16]. However, we identify deregulation not only of cytoplasmic translation but also protein targeting to the endoplasmic reticulum (ER) and plasma membrane. Changes in protein secretion may be biologically relevant especially in the human developing neocortex, which has a different extracellular matrix composition compared to rodents [30]. Notably, it has previously been reported that ER stress may protract the developmental shift from direct to indirect neurogenesis [70] and may thus explain the decreased proportion of TBR2-positive IPCs to SOX2-positive vRGs (Fig. 3). Finally, downregulation of translation machinery components may alter the translation landscape in a cell type-specific manner [71, 72]. We suggest that we can gain interesting mechanistic insights into cell type-specific translational changes induced by MIA in the future considering current developments in single-cell [73] and ultrasensitive [74] Ribo-seq.

Since gene expression has correlative structure where expression of groups of genes is driven by binding of TFs to the respective genomic loci, in so-called regulons, these regulons can be perceived as functional entities of gene expression [54]. Analysis of regulon activity in cycling vRGs revealed two major TFs driving transcriptional changes in Hyper-IL-6-treated organoids, STAT3 and NR2F1 (Fig. 5). STAT3 validates our model, since STAT3 is the known major transcription factor downstream of IL-6/JAK/STAT signaling [42]. In contrast, NR2F1 has not been reported to be involved in mediating the effects of MIA but it is an important TF with multiple cell type- and cortical/brain region-specific functions [75–77]. In dorsal forebrain RGs, it determines the balance between proliferation and differentiation by acting as a counterbalance to PAX6 [77]. Given that in comparison with murine data we do not observe dExNs overproduction in response to MIA, we hypothesize that *NR2F1* is upregulated in RGs as a protective factor against the neotenic effect of IL-6 and that this effect may differentiate mouse and human model systems. However, NR2F1 is also known as prominent modulator of neuronal migration [76] and, therefore, deregulation of NR2F1 in Hyper-IL-6-treated organoid may be causal for the observed defects in cortical lamination 35 days after treatment.

Taken together, our findings corroborate a causal role of abnormalities in protein translation for the development of ASD-like phenotypes upon MIA [16]. Additionally, the upregulation of genes involved in the innate immune response, including those of MHCI, partially recapitulates the effects of IFN-γ exposure in NPCs [22]. Therefore, our model demonstrates the utility of DFOs for investigating the molecular mechanisms underlying the effects of MIA on human neurodevelopment. While our study focused only on male DFOs, future studies may determine whether similar effects are seen in female DFOs or whether female DFOs possess inherent resilience mechanisms as suggested by mouse models [16].

In conclusion, our study established a model of MIA in brain region-specific organoids and provides a framework for future studies of the effects of MIA on multiple organizational levels, from subcellular to tissue-wide. Our results corroborate previous results from rodent and 2D in vitro models [16, 18, 22, 44]. However, we also extend our knowledge of cell types primarily affected by MIA, by showing that vRGs are specifically vulnerable to IL-6 signaling, shifting them towards a younger pseudoage along the neural differentiation trajectory. Finally, the DFO model of MIA may serve as a platform for investigating the effects of the interaction of genetic and environmental mediators of complex neurodevelopmental diseases such as ASD [36, 78, 79].

### Supplementary information

Supplementary methods

Figure S1

Figure S2

Figure S3

Figure S4

Figure S5

Supplementary Fig. legends

Supplementary Tables 1-16

## Data Availability

Sequencing data generated in this study will be accessible at the repository Array Express under accession numbers E-MTAB-12702 and E-MTAB-12743.

## References

[1] Faa G, Manchia M, Pintus R, Gerosa C, Marcialis MA, Fanos V (2016). Fetal programming of neuropsychiatric disorders. Birth Defects Res Part C: Embryo Today: Rev.

[2] Ekblad M, Korkeila J, Parkkola R, Lapinleimu H, Haataja L, Lehtonen L (2010). Maternal smoking during pregnancy and regional brain volumes in preterm infants. J Pediatr.

[3] Baron-Cohen S, Auyeung B, Norgaard-Pedersen B, Hougaard DM, Abdallah MW, Melgaard L (2015). Elevated fetal steroidogenic activity in autism. Mol Psychiatry.

[4] Auyeung B, Lombardo MV, Baron-Cohen S (2013). Prenatal and postnatal hormone effects on the human brain and cognition. Pflug Arch.

[5] Atladóttir HÓ, Thorsen P, Østergaard L, Schendel DE, Lemcke S, Abdallah M (2010). Maternal infection requiring hospitalization during pregnancy and autism spectrum disorders. J Autism Dev Disord.

[6] Brown AS (2012). Epidemiologic studies of exposure to prenatal infection and risk of schizophrenia and autism. Dev Neurobiol.

[7] Massrali A, Adhya D, Srivastava DP, Baron-Cohen S, Kotter MR. Virus-induced maternal immune activation as an environmental factor in the etiology of autism and schizophrenia. Front Neurosci. 2022;16:834058. 10.3389/fnins.2022.834058 PMC9039720 35495047

[8] Rudolph MD, Graham AM, Feczko E, Miranda-Dominguez O, Rasmussen JM, Nardos R (2018). Maternal IL-6 during pregnancy can be estimated from newborn brain connectivity and predicts future working memory in offspring. Nat Neurosci.

[9] Rasmussen JM, Graham AM, Entringer S, Gilmore JH, Styner M, Fair DA (2019). Maternal Interleukin-6 concentration during pregnancy is associated with variation in frontolimbic white matter and cognitive development in early life. Neuroimage.

[10] Spann MN, Monk C, Scheinost D, Peterson BS (2018). Maternal immune activation during the third trimester is associated with neonatal functional connectivity of the salience network and fetal to toddler behavior. J Neurosci.

[11] Smith SE, Li J, Garbett K, Mirnics K, Patterson PH (2007). Maternal immune activation alters fetal brain development through interleukin-6. J Neurosci.

[12] Meyer U, Nyffeler M, Engler A, Urwyler A, Schedlowski M, Knuesel I (2006). Th. J Neurosci.

[13] Careaga M, Murai T, Bauman MD (2017). Maternal immune activation and autism spectrum disorder: from rodents to nonhuman and human primates. Biol Psychiatry.

[14] Machado CJ, Whitaker AM, Smith SE, Patterson PH, Bauman MD (2015). Maternal immune activation in nonhuman primates alters social attention in juvenile offspring. Biol Psychiatry.

[15] Oskvig DB, Elkahloun AG, Johnson KR, Phillips TM, Herkenham M (2012). Maternal immune activation by LPS selectively alters specific gene expression profiles of interneuron migration and oxidative stress in the fetus without triggering a fetal immune response. Brain Behav Immun.

[16] Kalish BT, Kim E, Finander B, Duffy EE, Kim H, Gilman CK (2021). Maternal immune activation in mice disrupts proteostasis in the fetal brain. Nat Neurosci.

[17] Choi GB, Yim YS, Wong H, Kim S, Kim H, Kim SV (2016). The maternal interleukin-17a pathway in mice promotes autism-like phenotypes in offspring. Science.

[18] Ben-Reuven L, Reiner O (2021). Dynamics of cortical progenitors and production of subcerebral neurons are altered in embryos of a maternal inflammation model for autism. Mol Psychiatry.

[19] Mirabella F, Desiato G, Mancinelli S, Fossati G, Rasile M, Morini R (2021). Prenatal interleukin 6 elevation increases glutamatergic synapse density and disrupts hippocampal connectivity in offspring. Immunity.

[20] Mueller FS, Scarborough J, Schalbetter SM, Richetto J, Kim E, Couch A (2021). Behavioral, neuroanatomical, and molecular correlates of resilience and susceptibility to maternal immune activation. Mol Psychiatry.

[21] Sarieva K, Mayer S (2021). The effects of environmental adversities on human neocortical neurogenesis modeled in brain organoids. Front Mol Biosci.

[22] Warre-Cornish K, Perfect L, Nagy R, Duarte RRR, Reid MJ, Raval P (2020). Interferon-gamma signaling in human iPSC-derived neurons recapitulates neurodevelopmental disorder phenotypes. Sci Adv.

[23] Corsini NS, Knoblich JA (2022). Human organoids: New strategies and methods for analyzing human development and disease. Cell.

[24] Rose-John S. Interleukin-6 Family Cytokines. Cold Spring Harb Perspect Biol. 2018;10:a028415. 10.1101/cshperspect.a028415 PMC5793756 28620096

[25] Schumacher N, Meyer D, Mauermann A, Von Der Heyde J, Wolf J, Schwarz J (2015). Shedding of endogenous Interleukin-6 Receptor (IL-6R) is governed by A Disintegrin and Metalloproteinase (ADAM) Proteases while a Full-length IL-6R isoform localizes to circulating microvesicles. J Biol Chem.

[26] Couch ACM, Solomon S, Marrocu A, Duarte R, Sun Y, Sichlinger L, et al. Acute IL-6 exposure triggers canonical IL6Ra signaling in hiPSC microglia, but not neural progenitor cells. Brain Behav Immun. 2023;110:43–59. 10.1016/j.bbi.2023.02.007 PMC10682389 36781081

[27] Fischer M, Goldschmitt J, Peschel C, Brakenhoff JP, Kallen KJ, Wollmer A (1997). I. A bioactive designer cytokine for human hematopoietic progenitor cell expansion. Nat Biotechnol.

[28] Velmeshev D, Schirmer L, Jung D, Haeussler M, Perez Y, Mayer S (2019). Single-cell genomics identifies cell type–specific molecular changes in autism. Science.

[29] Hatta T, Moriyama K, Nakashima K, Taga T, Otani H (2002). The Role of gp130 in cerebral cortical development: in vivofunctional analysis in a MouseExo UteroSystem. J Neurosci.

[30] Pollen AA, Nowakowski TJ, Chen J, Retallack H, Sandoval-Espinosa C, Cory (2015). Molecular identity of human outer radial glia during cortical development. Cell.

[31] Nowakowski TJ, Bhaduri A, Pollen AA, Alvarado B, Mostajo-Radji MA, Di Lullo E (2017). Spatiotemporal gene expression trajectories reveal developmental hierarchies of the human cortex. Science.

[32] La Manno G, Siletti K, Furlan A, Gyllborg D, Vinsland E, Mossi Albiach A (2021). Molecular architecture of the developing mouse brain. Nature.

[33] Dahlgren J, Samuelsson A-M, Jansson T, Holmäng A (2006). Interleukin-6 in the maternal circulation reaches the rat fetus in mid-gestation. Pediatr Res.

[34] Velasco S, Kedaigle AJ, Simmons SK, Nash A, Rocha M, Quadrato G (2019). Individual brain organoids reproducibly form cell diversity of the human cerebral cortex. Nature.

[35] Mayer S, Chen J, Velmeshev D, Mayer A, Eze UC, Bhaduri A (2019). Multimodal single-cell analysis reveals physiological maturation in the developing human neocortex. Neuron.

[36] Loomes R, Hull L, Mandy WPL (2017). What Is the male-to-female ratio in autism spectrum disorder? A systematic review and meta-analysis. J Am Acad Child Adolesc Psychiatry.

[37] Blair JD, Hockemeyer D, Bateup HS (2018). Genetically engineered human cortical spheroid models of tuberous sclerosis. Nat Med.

[38] Stuart JM, Segal E, Koller D, Kim SK (2003). A Gene-Coexpression Network for Global Discovery of Conserved Genetic Modules. Science.

[39] Khakipoor S, Crouch EE, Mayer S (2020). Human organoids to model the developing human neocortex in health and disease. Brain Res.

[40] Qiu X, Li J, Bonenfant J, Jaroszewski L, Mittal A, Klein W, et al. Dynamic changes in human single‐cell transcriptional signatures during fatal sepsis. J Leukoc Biol. 2021;110:1253–68. 10.1002/JLB.5MA0721-825R PMC8629881 34558746

[41] Gabay C (2006). Interleukin-6 and chronic Inflammation. Arthritis Res Ther.

[42] Deverman BE, Patterson PH (2009). Cytokines and CNS Development. Neuron.

[43] Yoshimatsu T, Kawaguchi D, Oishi K, Takeda K, Akira S, Masuyama N (2006). Non-cell-autonomous action of STAT3 in maintenance of neural precursor cells in the mouse neocortex. Development.

[44] Lombardo MV, Moon HM, Su J, Palmer TD, Courchesne E, Pramparo T (2018). Maternal immune activation dysregulation of the fetal brain transcriptome and relevance to the pathophysiology of autism spectrum disorder. Mol Psychiatry.

[45] Kang Y, Zhou Y, Li Y, Han Y, Xu J, Niu W (2021). A human forebrain organoid model of fragile X syndrome exhibits altered neurogenesis and highlights new treatment strategies. Nat Neurosci.

[46] Voineagu I, Wang X, Johnston P, Lowe JK, Tian Y, Horvath S (2011). Transcriptomic analysis of autistic brain reveals convergent molecular pathology. Nature.

[47] Tanaka Y, Cakir B, Xiang Y, Sullivan GJ, Park I-H (2020). Synthetic analyses of single-cell transcriptomes from multiple brain organoids and fetal brain. Cell Rep.

[48] Zhong S, Zhang S, Fan X, Wu Q, Yan L, Dong J (2018). A single-cell RNA-seq survey of the developmental landscape of the human prefrontal cortex. Nature.

[49] Fleck JS, Sanchis-Calleja F, He Z, Santel M, Boyle MJ, Camp JG (2021). Resolving organoid brain region identities by mapping single-cell genomic data to reference atlases. Cell Stem Cell.

[50] Thompson CL, Ng L, Menon V, Martinez S, Lee CK, Glattfelder K (2014). A high-resolution spatiotemporal atlas of gene expression of the developing mouse brain. Neuron.

[51] Miller JA, Ding S-L, Sunkin SM, Smith KA, Ng L, Szafer A (2014). Transcriptional landscape of the prenatal human brain. Nature.

[52] Lambert SA, Jolma A, Campitelli LF, Das PK, Yin Y, Albu M (2018). The human transcription factors. Cell.

[53] Satterstrom FK, Kosmicki JA, Wang J, Breen MS, De Rubeis S, An J-Y (2020). Large-scale exome sequencing study implicates both developmental and functional changes in the neurobiology of autism. Cell.

[54] Aibar S, González-Blas CB, Moerman T, Huynh-Thu VA, Imrichova H, Hulselmans G (2017). SCENIC: single-cell regulatory network inference and clustering. Nat Methods.

[55] Suo S, Zhu Q, Saadatpour A, Fei L, Guo G, Yuan G-C (2018). Revealing the critical regulators of cell identity in the mouse cell atlas. Cell Rep.

[56] Allswede DM, Yolken RH, Buka SL, Cannon TD (2020). Cytokine concentrations throughout pregnancy and risk for psychosis in adult offspring: a longitudinal case-control study. Lancet Psychiatry.

[57] Rakemann T, Niehof M, Kubicka S, Fischer M, Manns MP, Rose-John S (1999). The designer cytokine Hyper-Interleukin-6 is a potent activator of STAT3-dependent gene transcription in vivo and in vitro. J Biol Chem.

[58] Popova S, Lange S, Probst C, Gmel G, Rehm J (2017). Estimation of national, regional, and global prevalence of alcohol use during pregnancy and fetal alcohol syndrome: a systematic review and meta-analysis. Lancet Glob Health.

[59] Fagerlund I, Dougalis A, Shakirzyanova A, Gomez-Budia M, Pelkonen A, Konttinen H, et al. Microglia-like Cells Promote Neuronal Functions in Cerebral Organoids. Cells 2020;11:124. 10.3390/cells11010124 PMC8750120 35011686

[60] Abud EM, Ramirez RN, Martinez ES, Healy LM, Nguyen CHH, Newman SA (2017). iPSC-derived human microglia-like cells to study neurological diseases. Neuron.

[61] Smart IHM, Dehay C, Giroud P, Berland M, Kennedy H (2002). Unique morphological features of the proliferative zones and postmitotic compartments of the neural epithelium giving rise to striate and extrastriate cortex in the monkey. Cereb Cortex.

[62] Fietz SA, Kelava I, Vogt J, Wilsch-Bräuninger M, Stenzel D, Fish JL (2010). OSVZ progenitors of human and ferret neocortex are epithelial-like and expand by integrin signaling. Nat Neurosci.

[63] Hansen DV, Lui JH, Parker PRL, Kriegstein AR (2010). Neurogenic radial glia in the outer subventricular zone of human neocortex. Nature.

[64] Cebrián C, Loike JD, Sulzer D. Neuronal MHC-I expression and its implications in synaptic function, axonal regeneration and Parkinson’s and other brain diseases. Front Neuroanat. 2014;8:00114. 10.3389/fnana.2014.00114 PMC4195363 25352786

[65] Kathuria A, Nowosiad P, Jagasia R, Aigner S, Taylor RD, Andreae LC (2018). Stem cell-derived neurons from autistic individuals with SHANK3 mutation show morphogenetic abnormalities during early development. Mol Psychiatry.

[66] Deshpande A, Yadav S, Dao DQ, Wu Z-Y, Hokanson KC, Cahill MK (2017). Cellular phenotypes in human iPSC-derived neurons from a genetic model of autism spectrum disorder. Cell Rep.

[67] Schafer ST, Paquola ACM, Stern S, Gosselin D, Ku M, Pena M (2019). Pathological priming causes developmental gene network heterochronicity in autistic subject-derived neurons. Nat Neurosci.

[68] Elmer BM, Estes ML, Barrow SL, McAllister AK (2013). MHCI requires MEF2 transcription factors to negatively regulate synapse density during development and in disease. J Neurosci.

[69] Al-Hakbany M, Awadallah S, Al-Ayadhi L (2014). The relationship of HLA Class I and II Alleles and Haplotypes with autism: a case-control study. Autism Res Treat.

[70] Godin JD, Creppe C, Laguesse S, Nguyen L (2016). Emerging roles for the unfolded protein response in the developing nervous system. Trends Neurosci.

[71] Harnett D, Ambrozkiewicz MC, Zinnall U, Rusanova A, Borisova E, Drescher AN, et al. A critical period of translational control during brain development at codon resolution. Nat Struct Mol Biol. 2022;29:1277–90. 10.1038/s41594-022-00882-9 PMC9758057 36482253

[72] Ambrozkiewicz MC, Borisova E, Newman AG, Kraushar ML, Schaub T, Dannenberg R, et al. Ire1α-regulated mRNA translation rate controls the identity and polarity of upper layer cortical neurons. bioRxiv 2022.

[73] VanInsberghe M, van den Berg J, Andersson-Rolf A, Clevers H, van Oudenaarden A (2021). Single-cell Ribo-seq reveals cell cycle-dependent translational pausing. Nature.

[74] Xiong Z, Xu K, Lin Z, Kong F, Wang Q, Quan Y (2022). Ultrasensitive Ribo-seq reveals translational landscapes during mammalian oocyte-to-embryo transition and pre-implantation development. Nat Cell Biol.

[75] Del Pino I, Tocco C, Magrinelli E, Marcantoni A, Ferraguto C, Tomagra G (2020). COUP-TFI/Nr2f1 orchestrates intrinsic neuronal activity during development of the somatosensory cortex. Cereb Cortex.

[76] Tocco C, Bertacchi M, Studer M. Structural and functional aspects of the neurodevelopmental gene NR2F1: From animal models to human pathology. Front Mol Neurosci. 2021;14:767965. 10.3389/fnmol.2021.767965 PMC8715095 34975398

[77] Bertacchi M, Romano AL, Loubat A, Tran Mau‐Them F, Willems M, Faivre L, et al. NR2F1 regulates regional progenitor dynamics in the mouse neocortex and cortical gyrification in BBSOAS patients. EMBO J. 2020;39:e104163. 10.15252/embj.2019104163 PMC7327499 32484994

[78] Paulsen B, Velasco S, Kedaigle AJ, Pigoni M, Quadrato G, Deo A, et al. Autism genes converge on asynchronous development of shared neuron classes. Nature 2022;602:268–73. 10.1038/s41586-021-04358-6 PMC8852827 35110736

[79] Jourdon A, Wu F, Mariani J, Capauto D, Norton S, Tomasini L, et al. ASD modelling in organoids reveals imbalance of excitatory cortical neuron subtypes during early neurogenesis. bioRxiv 2022. 10.1038/s41593-023-01399-0 PMC10573709 37563294

[80] Rasmussen Mikkel A, Holst B, Tümer Z, Johnsen Mads G, Zhou S, Stummann Tina C (2014). Transient p53 suppression increases reprogramming of human fibroblasts without affecting apoptosis and DNA damage. Stem Cell Rep.

