# Supplementary material for: Human brain organoid model of maternal immune activation identifies radial glia cells as selectively vulnerable
Source: Mol Psychiatry. 2023 Mar 6;28(12):5077–89. doi: 10.1038/s41380-023-01997-1 (PMC9986664; doi:10.1038/s41380-023-01997-1)
Supplement: Supplementary file 1 — Supplementary methods [file 41380_2023_1997_MOESM1_ESM.pdf]

Supplementary Materials and Methods

iPS cell culture and dorsal forebrain organoids differentiation

Human iPSC lines BIONi010-C (Source: EBiSC) and HMGU1 (Source: Human Pluripotent Stem Cell Registry) were cultured in standard conditions (37°C, 5% CO_2, and 100% humidity), in E8 flex medium (Gibco, Cat. no. A2858501) and passaged in colonies using Gentle Dissociation Reagent (STEMCELL Technologies, Cat. no. 07174) onto hESC-qualified growth factor-reduced Matrigel-coated (Corning, Cat. no. 354277) plates. Cell lines were maintained below passage 25, and were routinely tested for mycoplasma contamination (assayed with PCR Mycoplasma Detection Set, TaKaRa, Cat. no. 6601) and pluripotency status with antibodies against OCT4 (rabbit, 1:500, Abcam, Cat. no. ab19857) upon thawing each cryovial.

Dorsal forebrain organoids were generated as previously described with minor modifications (1). Briefly, iPSCs at ~70-80% confluency were dissociated into single cells using Accutase (MERCK, Cat. no. A6964) and plated at 9 000 cells per well in 96 well V-bottom low adhesion plates (S-bio, Cat. no. MS-9096VZ) in cortical differentiation medium I (CDMI) supplemented with 20 µM Y-27632 (Cayman Chemical, Cat. no. 10005583), 5 µM SB 431542 (Tocris, Cat. no. 1614), and 3 µM IWR-1 (MERCK, Cat. no. 681669). Glasgow's MEM (GMEM)-based (Gibco, Cat. no. 11710035) CDMI includes 20% KnockOut Serum Replacement (KOSR, Thermofisher Scientific, Cat. no. 10828028), 1X non-essential amino acids (Sigma, Cat. no. M7145), 0.11 mg/mL Sodium Pyruvate (Thermofisher Scientific, Cat. no. 11360070), 1X Penicillin-Streptomycin (Thermofisher Scientific, Cat. no. 15140122), 0.1 mM β-Mercaptoethanol (Gibco, Cat. no. 21985023). At this stage, media was changed every three days. After six days of differentiation, Y-27632 was withdrawn by media change. After 18 days, dorsal forebrain organoids were transferred from 96- to 24-well low adhesion plates and moved to an orbital shaker rotating at 53 rpm (2.5 cm throw) with media exchange to CDMII (DMEM/F12-based medium containing 1X Glutamax (Thermofisher Scientific, Cat. no. 31331093), 1X N2 Supplement

(Thermofisher Scientific, Cat. no. 17502048), 1X CD Lipid Concentrate (Thermofisher Scientific, Cat. no. 11905031) and 1X Penicillin-Streptomycin (Thermofisher Scientific, Cat. no. 15140122)). At 35 days, organoids were moved into CDMIII (DMEM/F12-based medium containing 10% FBS (GE Healthcare Life Sciences, Cat. no. SH30070.03), 5 µg/mL Heparin (MERCK, Cat. no. H3149-25KU), 1X N2 Supplement, 1X CD Lipid Concentrate and 1% Matrigel (Corning, Cat. no. 356234)). At 70 days, CDMIV was introduced by additionally supplementing CDMIII with 1X B27 Supplement (Thermofisher Scientific, Cat. no. 17504044). Starting from day 18, media changes were performed every 3-4 days.

To treat organoids with IL-6 and Hyper-IL-6, the compounds were diluted in 0.1% BSA (AppliChem, Cat. no. A0850) in PBS (also used as Vehicle control). At the start of treatment, a complete media change was performed with CDMIII containing respective compounds at a final concentration of 8.8 ng/ml (422.6 nM) IL-6 (R&D Systems, Cat. no. 7270-IL-025) and 25 ng/ml (422.6 nM) Hyper-IL-6 (R&D Systems, Cat. no. 8954-SR-025) at day 45 of differentiation. The treatment was maintained by daily half-volume media change until termination of the experiment or treatment withdrawal at day 55 of differentiation.

Immunohistochemistry in organoid sections and human tissue samples

Whole dorsal forebrain organoids were fixed in 4% paraformaldehyde (PFA) in PBS for 45–60 min at room temperature (2). Organoids were washed three times with PBS and then incubated in 30% sucrose in PBS solution until saturated. Organoids were embedded in a 1:1 v:v mixture of 30% sucrose in PBS and optimal cutting temperature (OCT) compound (TissueTek) and sectioned on Superfrost Plus slides (VWR) with a cryostat at 20 µm (Leica).

For immunostaining, slides were thawed to room temperature, washed with PBS before permeabilization and blocking with 1% Triton-X100, 0.2% gelatin, and 10% normal donkey serum in PBS for 1 hour. The following primary antibodies were used: anti-Ki-67 (rabbit, 1:300, Merck, Cat. no. AB9260), anti-SOX2 (goat, 1:500, R&D Systems, Cat. no. AF2018), anti-CTIP2 (rat, 1:400, Abcam, Cat. no. ab18465), anti-TBR2 (rabbit, 1:100, Atlas Antibodies, HPA028896), anti-SATB2 (rabbit, 1:500,

Abcam, ab34735), anti-PPP1R17 (rabbit, 1:1000, Atlas Antibodies, HPA047819), anti-IL6ST (goat, 1:100, R&D Systems, AF-228-NA), anti-Nestin (rabbit, 1:500, Merck, ABD69), anti-p-Y705-STAT3 (rabbit, 1:100-200, Cell Signaling Technologies, Cat. no. 9145), anti-NR2F1 (rabbit, 1:100, Abcam, Cat. no. ab181137). For immunostainings with anti-TBR2, anti- p-Y705-STAT3 and anti-NR2F1 antibodies, antigen retrieval in boiling 10 mM citric acid buffer (pH 6.0) was performed. Primary antibodies diluted in permeabilization and blocking solution were applied to the sections overnight at 4°C. After washing three times with PBS, secondary antibodies diluted in permeabilization and blocking solution were applied to the sections for 3 hours at room temperature. Finally, sections were washed three times with PBS and stained with the nuclear dyes DAPI (Thermofisher Scientific, Cat. no. D1306) or DRAQ5 (Thermofisher Scientific, Cat. no. 62251) following the respective protocols supplied by the manufacturer. Images were acquired by Leica SP8 confocal microscope at 40x magnification objective. Quantitative analyses were conducted on three or more randomly selected rosette structures per section of an organoid unless stated otherwise. The numbers of cells positive for each marker were measured with Imaris (v.9.7) software using "Surfaces" and "Spots" options (Bitplane, RRID:SCR_007370). For quantitative analysis of Ki-67-, TBR2- and SOX2-positive cells the following procedure was performed. First, the ventricular zone (VZ)-like regions were selected based on the SOX2 reactivity and dense localization in neural rosettes. Second, SOX2-positive cells within rosettes were called "ventricular" and their number was quantified. Ki-67-positive cells were quantified within VZs and therefore these cells represent proliferative population within VZ. TBR2-positive cells were counted from regions adjacent to VZ-like areas which represent putative subventricular zone (SVZ). Both weakly- and strongly-TBR2-expressing cells were quantified. To quantify the area, perimeter and number of VZ-like regions, the VZ was defined by dense SOX2+ immunoreactivity from epifluorescent images. Several sections (4 for D50, 6 for D55) sampled at equal intervals (120 µm) of each organoid were quantified and the results were averaged across sections prior to further analysis. To quantify the distribution of CTIP2+/SATB2+ cells, the cell numbers were quantified in 200 µm-wide regions of interest starting from the outer surface of the organoid (three randomly chosen positions on the section of the organoid). The inner border of putative cortical plate (CP) was defined

by the end of CTIP2 reactivity. Their y-coordinates of individual CTIP2- and SATB2-positive cells were recorded and normalized to the range of y-coordinates per image (Min-max normalization) to measure their relative laminar positions in the CP. The frequency distributions of the relative positions in 10 evenly divided bins for each marker were calculated and plotted in R. The CTIP2- and SATB2- co-expression was measured using Matlab "Colocalize spots" plugin as implemented in Imaris software. The ratio of double-positive cells to single-positive cells was calculated by [#CTIP2+ SATB2+ nuclei/#(SATB2+ or CTIP2+) nuclei]. For all immunohistochemistry samples, the images were excluded from analysis if immunostaining quality was poor or uneven.

Western Blotting

Organoids were lysed in 1X RIPA buffer (Cell Signaling technology, Cat. no. 9806) in the presence of 1x Halt protease and phosphatase inhibitor cocktail (Thermofisher Scientific, Cat. no. 78443) (3). Subsequently, the samples were incubated on rotating wheel for 1 hour, sonicated using probe sonicator (20 seconds, 10 pulses, 50% power, Bandelin, SONOPULS mini20, MS1.5 probe) and centrifuged at 14 000 g for 30 minutes at 4°C. The volume of resulting supernatant was reduced using centrifugal filters Amicon Ultra 3K (Sigma, Cat.no. UFC500396) at 14 000 g for 30 minutes at 4°C. The total protein concentrations were determined using the Pierce™ BCA assay (Thermofisher Scientific, Cat. no. 23225). Samples containing equal amounts of protein (5-10 µg) were boiled in complete sample buffer (Li-COR, Cat. no. 928-40004) at either 95°C for 5 minutes or at 70°C for 10 minutes and resolved in a 4-15% Precast Gels (Bio-Rad, 12-well, Cat. no. 456-1095, 15-well, Cat. no. 456-1086). Proteins were transferred onto Immun-Blot Low Fluorescence PVDF membrane (Bio-Rad, Cat. no. 1620264) using Trans-blot Turbo (Bio-Rad) semi-dry transfer system. Total protein staining in membranes was performed using Revert™ 700 Total Protein Stain Kit for Western Blot Normalization (Li-COR, Cat. no. 926-11016). The membranes were blocked with Tris-buffered saline-based blocking buffer (Li-COR, Cat. no. 927-60001) for 1 hour at room temperature. Subsequently, the membranes were incubated with the primary antibodies diluted in Tris-buffered saline-based antibody dilution buffer (Li-COR, Cat. no. 927-65001) at 4°C overnight. The following primary antibodies were used:

anti-STAT3 (mouse, 1:1000, Cell Signaling Technologies, Cat. no. 9139), anti-p-Y705-STAT3 (rabbit, 1:1000, Cell Signaling Technologies, Cat. no. 9145), anti-NR2F1 (rabbit, 1:1000, Abcam, Cat. no. ab181137) and anti-β-actin (mouse, 1:5000, Abcam, Cat. no. ab6276). After washing in 1X TBS-T (Tris-buffered saline pH 7.5, 0.1% Tween-20), the membranes were incubated with anti-rabbit (Li-COR, Cat. no. 926-32211) and anti-mouse (Li-COR, Cat. no. 926-32211) secondary antibody conjugated with infra-red dyes for 1 hour at room temperature. Blots were then imaged with Odyssey Scanner and analyzed using Image Studio and Image Studio Lite v.5 software (Li-COR) with normalization to the background and Total Protein Stain. For all Western blot analyses, the samples were excluded if the protein transfer was uneven or there were air bubbles trapped between the membrane and the gel.

RNA sequencing

mRNA was isolated from organoids using the RNeasy Mini Kit (Qiagen, Cat. no. 74104) and RNase-Free DNase set (Qiagen, Cat. no. 79254). Library preparation was conducted by Novogene (https://en.novogene.com/) using non-directional poly-A enrichment strategy. Samples were then sequenced on the Illumina NovaSeq 6000 platform using paired-end, 150-bp-long reads to 30 million reads per sample. HISAT2 was used to align raw reads to the GRCh37/hg19 human genome reference (4). Reads containing adaptors, more than 10% of undefined bases and more than 50% of bases with Qscore (Quality value) <= 5 were excluded from further analysis. Gene expression levels were estimated using FPKM (expected number of Fragments Per Kilobase of transcript sequence per Millions base pairs sequenced) (5). Principal component analysis (PCA) was performed on the FPKM values. Hierarchical clustering was used to cluster the FPKM values of genes. Differential gene expression (DGE) analysis was performed using the DESeq2 R package (v.1.10.1) (6). P-values were adjusted for multiple comparisons using the Benjamini-Hochberg method and significantly differentially expressed genes were selected based on two criteria: FDR ≤ 0.2 and absolute log2 Fold Change > 0.4 between the Vehicle and Hyper-IL-6 conditions. Functional analysis of the differentially expressed genes was performed using clusterProfiler (v.3.18.1) (7). Weighted gene co-expression

analysis (WGCNA) was performed using normalized FPKM values. The WGCNA R package computed an unsigned similarity matrix with a softpower of 10 (8). Gene modules were created on the basis of hierarchical clustering results of the similarity matrix and eigengene per module was calculated. The relationship between gene modules and experimental conditions was evaluated by comparing eigengene values by two-sided t-test with a Bonferroni correction for multiple comparisons. Network reconstruction for the light-yellow module was performed using Cytoscape (v3.9.1) and nodes were colored based on their functional annotation using gene set overrepresentation analysis or their enrichment in the radial glia cells based on the gene lists from Pollen and colleagues (9). The majority of bioinformatics analysis was conducted by Novogene (https://en.novogene.com/). No samples were excluded from the analysis based on the initial sample quality assessment.

Single-cell dissociation and multiplexing for single-cell RNA sequencing

Individual dorsal forebrain organoids were dissociated using the Worthington Papain Dissociation System kit (Worthington Biochemical, Cat. no. LK003150) following a published protocol (10) with minor modifications. Organoids were cut with a scalpel blade and then placed in 2.5 ml papain supplemented with DNAse inhibitor and incubated at 37 °C for 15 min while shaking at 57 rpm (2.5 cm throw). Organoids were gently triturated ten times using a 10-ml serological pipette and a 1-ml pipette, and incubation continued for another 10 min. Next, organoids were triturated ten times using a 10-ml serological pipette. Large debris was allowed to settle down in the Ovomucoid Inhibitor diluted in Earle's medium, and the supernatant containing single cells was centrifuged at 300 g for 7 minutes at room temperature. Cells were resuspended in 0.04% BSA in PBS and passed through a 40-µm cell strainer (Flowmi). Dead Cell Removal (Miltenyi, Cat. no. 130-090-101) was performed following the manufacturer's instructions. After final centrifugation, cells were subjected to multiplexing using 3' CellPlex Kit Set A (10x Genomics, Cat. no. PN-1000261) following the manufacturer's instructions. Finally, the cells were quantified and pooled in equal proportions. Approximately 16 500 cells per channel were loaded onto a Chromium Single Cell 3' Chip (10x Genomics, Cat. no. PN-120236) and processed through the Chromium controller to generate single-

cell gel beads in emulsion. scRNAseq libraries were prepared with the Chromium Single Cell 3' Library & Gel Bead Kit v.3 (10x Genomics, Cat. no. PN-1000121). Libraries from different samples were pooled and sequenced on a NovaSeq6000 instrument (Illumina).

Single-cell RNA sequencing analysis

Quality control and preprocessing

scRNAseq data were aligned to the GRCh38-0.1.2 reference genome, and single-cell gene read count tables were generated using the CellRanger (v.3.6.0) pipelines multi and aggr without normalization. Data were analyzed in R (v4.0) using Seurat (v.4.0.4) (11). Quality control removed cells with fewer than 1 000 and more than 7 500 genes per cell, cells with log10(genes per UMI) less than 0.8 and cells with greater than 25% mitochondrial content. Gene expression was normalized using the SCTransform workflow and the different samples were then integrated using the integration workflow as published (12). Dimensionality reduction was performed using PCA, and we selected 30 PCs based on the Elbow plot. Louvain clustering was performed at resolutions from 0.2 to 1.0, and resolution 0.4 was chosen based on clustree (v.0.4.4) cluster stability assessment (13). UMAP plots were generated with Seurat package default parameters. Doublets were removed using DoubletFinder (14). For identifying the markers for each cluster, the FindAllMarkers function was used with both MAST (15) and roc algorithms applied to the raw count matrix. Additionally, we manually assessed expression of canonical genes to assign each cluster to a known cell type. Brain regional identity of individual clusters was assessed using the VoxHunt algorithm and SCTransform expression values were used as input (16).

Cell proportion analysis

Differences in cell type proportions between experimental conditions were analyzed with a permutation test followed by bootstrapping (https://github.com/rpolicastro/scProportionTest) where clusters with FDR less than 0.05 and absolute log2 fold change more than 0.58 were considered differentially abundant.

Trajectory inference and trajectory quality assessment

Slingshot (v.1.8.0) algorithm was applied to construct putative single-cell differentiation trajectories based on the UMAP representation (17). Pseudotime zero was assigned to the vRG cell cluster. A random forest classifier was used to retrieve genes differentially expressed along the putative deep-layer neuron differentiation trajectory.

Identification of differentially expressed genes, gene module signatures and gene set enrichment analysis

To identify differentially expressed genes between experimental conditions by cell type, we performed a DGE analysis using DESeq2 as implemented in the Seurat package. To control for differences in cluster representation between the samples, a random selection of N cells per cluster was chosen where N was the minimal cell count for each cluster across samples. Genes with FDR < 0.05 were considered statistically significant. Gene set enrichment analysis was performed using the clusterProfiler R package (FDR < 0.05 for significant gene ontology terms) (7). DEGs were annotated as transcription factors based on (18).

To calculate differential expression of modules, SCTransform normalized count matrix was supplied to UCell (v.1.1.1) algorithm using a predefined gene list from selected GO terms (19).

Additional gene set enrichment analysis was performed for ASD risk genes from the SFARI database (categories 1-4 from SFARI release on 31.10.2019; categories 1-2 from SFARI release on 20.07.2022) (20). Enrichment was tested using a one-sided Fisher's exact test in R to test whether the proportion of risk genes in the differentially expressed set in each cell cluster is more than expected by chance. Genes expressed in our dataset as assessed by DESeq2 were used as background. Up- and down-regulated genes were tested simultaneously. P-values were corrected for multiple tests (by number of cell clusters) using the Benjamini-Hochberg method.

SCENIC analysis

A single-cell regulatory network for cycling vRGs was constructed with SCENIC (v.1.2.4) (21). Specifically, GENIE3 (v.1.12.0) was applied to infer gene regulatory networks from log-normalized count data (22). For identification and scoring gene regulatory networks, or regulons, AUC was calculated using AUCell (v.1.13.3). Regulon specificity scores were calculated based on (23).

Statistics

The data distribution was not assumed to be normal in most cases and, therefore, Aligned Rank Transform (ART) was used for statistical testing unless stated otherwise. The cell line of origin and batch of organoid differentiation was included as a covariate in the models unless stated otherwise. Organoids were randomized into different treatment groups with approximately equal numbers of organoids in each group whenever possible. Details of specific statistical comparisons are listed in the relevant figure legends. No formal comparison of variances was performed between experimental groups. No statistical methods were used to predetermine sample size. Sample size was determined based on the work of other groups using human iPSC-derived brain organoid experimental model systems. For the immunohistochemical experiments, data analyses were run in a semiblind way, which means that the person performing data pre-processing did not have any information about the hypothesis and experimental procedures. In the case of all other experiments, investigators were not blinded during group allocation and data analysis due to the nature of the experiments. The experiments were reproduced with 1 or 2 batches of organoids differentiation per iPSC line of origin as indicated in the respective figure legends.

Supplementary References

1. Velasco S, Kedaigle AJ, Simmons SK, Nash A, Rocha M, Quadrato G, et al. Individual brain organoids reproducibly form cell diversity of the human cerebral cortex. Nature. 2019(570):523–7.
2. Lancaster MA, Knoblich JA. Generation of cerebral organoids from human pluripotent stem cells. Nat Protoc. 2014;9(10):2329-40.
3. Blair JD, Hockemeyer D, Bateup HS. Genetically engineered human cortical spheroid models of tuberous sclerosis. Nature Medicine. 2018;24(10):1568-78.
4. Kim D, Paggi JM, Park C, Bennett C, Salzberg SL. Graph-based genome alignment and genotyping with HISAT2 and HISAT-genotype. Nature Biotechnology. 2019;37(8):907-15.
5. Mortazavi A, Williams BA, McCue K, Schaeffer L, Wold B. Mapping and quantifying mammalian transcriptomes by RNA-Seq. Nature Methods. 2008;5(7):621-8.
6. Love MI, Huber W, Anders S. Moderated estimation of fold change and dispersion for RNA-seq data with DESeq2. Genome Biology. 2014;15(12).
7. Yu G, Wang L-G, Han Y, He Q-Y. clusterProfiler: an R Package for Comparing Biological Themes Among Gene Clusters. OMICS: A Journal of Integrative Biology. 2012;16(5):284-7.
8. Langfelder P, Horvath S. WGCNA: an R package for weighted correlation network analysis. BMC Bioinformatics. 2008;9(1):559.
9. Pollen AA, Nowakowski TJ, Chen J, Retallack H, Sandoval-Espinosa C, Cory, et al. Molecular Identity of Human Outer Radial Glia during Cortical Development. Cell. 2015;163(1):55-67.
10. Velasco S, Paulsen B, Arlotta P. Highly reproducible human brain organoids recapitulate cerebral cortex cellular diversity. 2019.
11. Hao Y, Hao S, Andersen-Nissen E, Mauck WM, Zheng S, Butler A, et al. Integrated analysis of multimodal single-cell data. Cell. 2021;184(13):3573-87.e29.
12. Stuart T, Butler A, Hoffman P, Hafemeister C, Papalexi E, Mauck WM, et al. Comprehensive Integration of Single-Cell Data. Cell. 2019;177(7):1888-902.e21.
13. Zappia L, Oshlack A. Clustering trees: a visualization for evaluating clusterings at multiple resolutions. GigaScience. 2018;7(7).
14. McGinnis CS, Murrow LM, Gartner ZJ. DoubletFinder: Doublet Detection in Single-Cell RNA Sequencing Data Using Artificial Nearest Neighbors. Cell Systems. 2019;8(4):329-37.e4.
15. Finak G, McDavid A, Yajima M, Deng J, Gersuk V, Shalek AK, et al. MAST: a flexible statistical framework for assessing transcriptional changes and characterizing heterogeneity in single-cell RNA sequencing data. Genome Biology. 2015;16(1).
16. Fleck JS, Sanchis-Calleja F, He Z, Santel M, Boyle MJ, Camp JG, et al. Resolving organoid brain region identities by mapping single-cell genomic data to reference atlases. Cell Stem Cell. 2021;28(6):1177-80.
17. Street K, Risso D, Fletcher RB, Das D, Ngai J, Yosef N, et al. Slingshot: cell lineage and pseudotime inference for single-cell transcriptomics. BMC Genomics. 2018;19(1).
18. Lambert SA, Jolma A, Campitelli LF, Das PK, Yin Y, Albu M, et al. The Human Transcription Factors. Cell. 2018;172(4):650-65.
19. Andreatta M, Carmona SJ. UCell: Robust and scalable single-cell gene signature scoring. Computational and Structural Biotechnology Journal. 2021;19:3796-8.
20. Weiss L, Abrahams B, Arking D, Campbell D, Mefford H, Morrow E, et al. SFARI Gene 2.0: a community-driven knowledgebase for the autism spectrum disorders (ASDs). 2013.
21. Aibar S, González-Blas CB, Moerman T, Huynh-Thu VA, Imrichova H, Hulselmans G, et al. SCENIC: single-cell regulatory network inference and clustering. Nature Methods. 2017;14(11):1083-6.
22. Huynh-Thu VA, Irrthum A, Wehenkel L, Geurts P. Inferring Regulatory Networks from Expression Data Using Tree-Based Methods. PLoS ONE. 2010;5(9):e12776.
23. Suo S, Zhu Q, Saadatpour A, Fei L, Guo G, Yuan G-C. Revealing the Critical Regulators of Cell Identity in the Mouse Cell Atlas. Cell Reports. 2018;25(6):1436-45.e3.
