# Supplementary figures and images for: Human brain organoid model of maternal immune activation identifies radial glia cells as selectively vulnerable
Source: Mol Psychiatry. 2023 Mar 6;28(12):5077–89. doi: 10.1038/s41380-023-01997-1 (PMC9986664; doi:10.1038/s41380-023-01997-1)

# Supplementary Figure 1

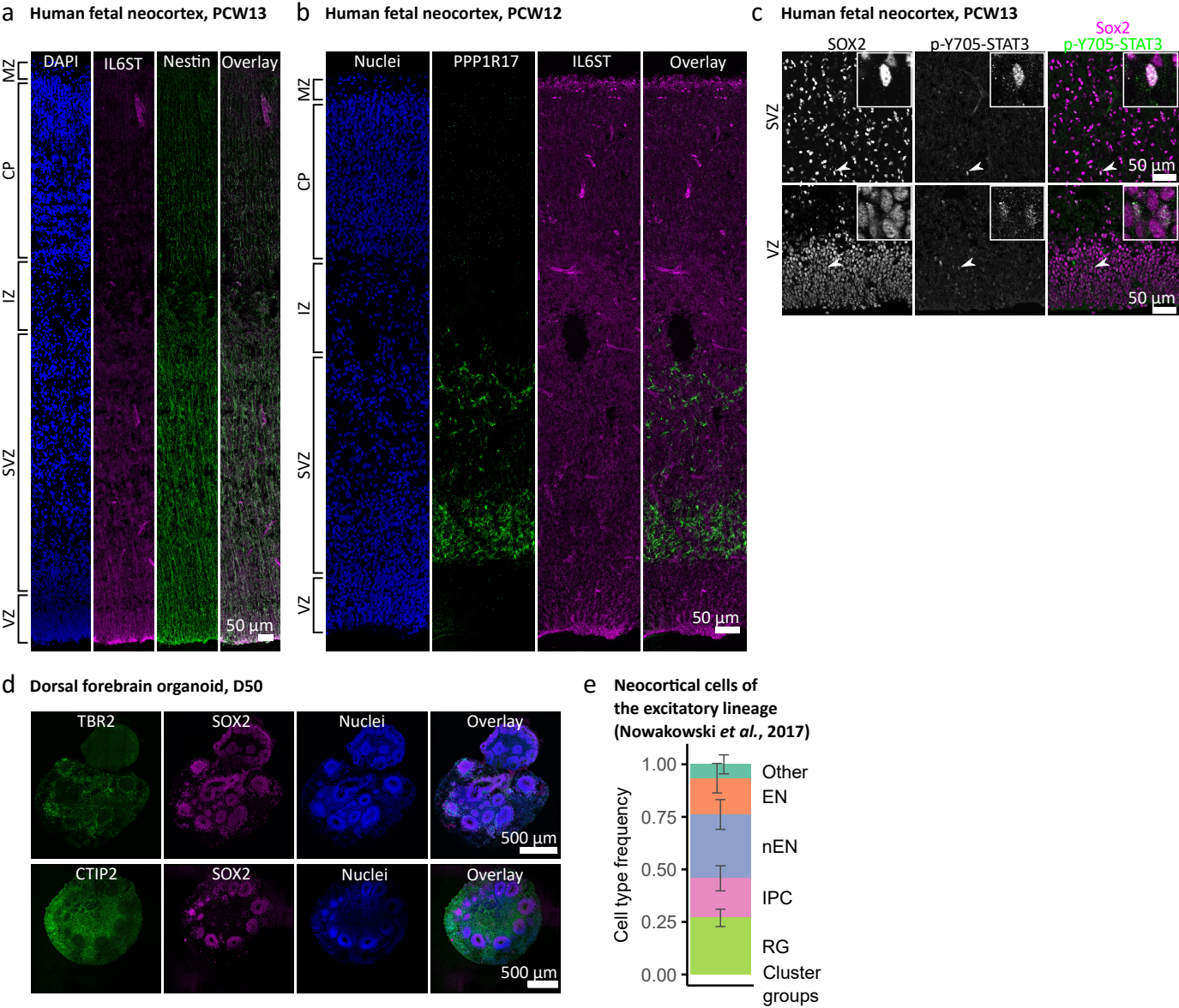

Supplement: Supplementary file 2 — Figure S1 [file 41380_2023_1997_MOESM2_ESM.pdf]
