# Supplementary material for: Human brain organoid model of maternal immune activation identifies radial glia cells as selectively vulnerable
Source: Mol Psychiatry. 2023 Mar 6;28(12):5077–89. doi: 10.1038/s41380-023-01997-1 (PMC9986664; doi:10.1038/s41380-023-01997-1)

Supplementary Figure 2

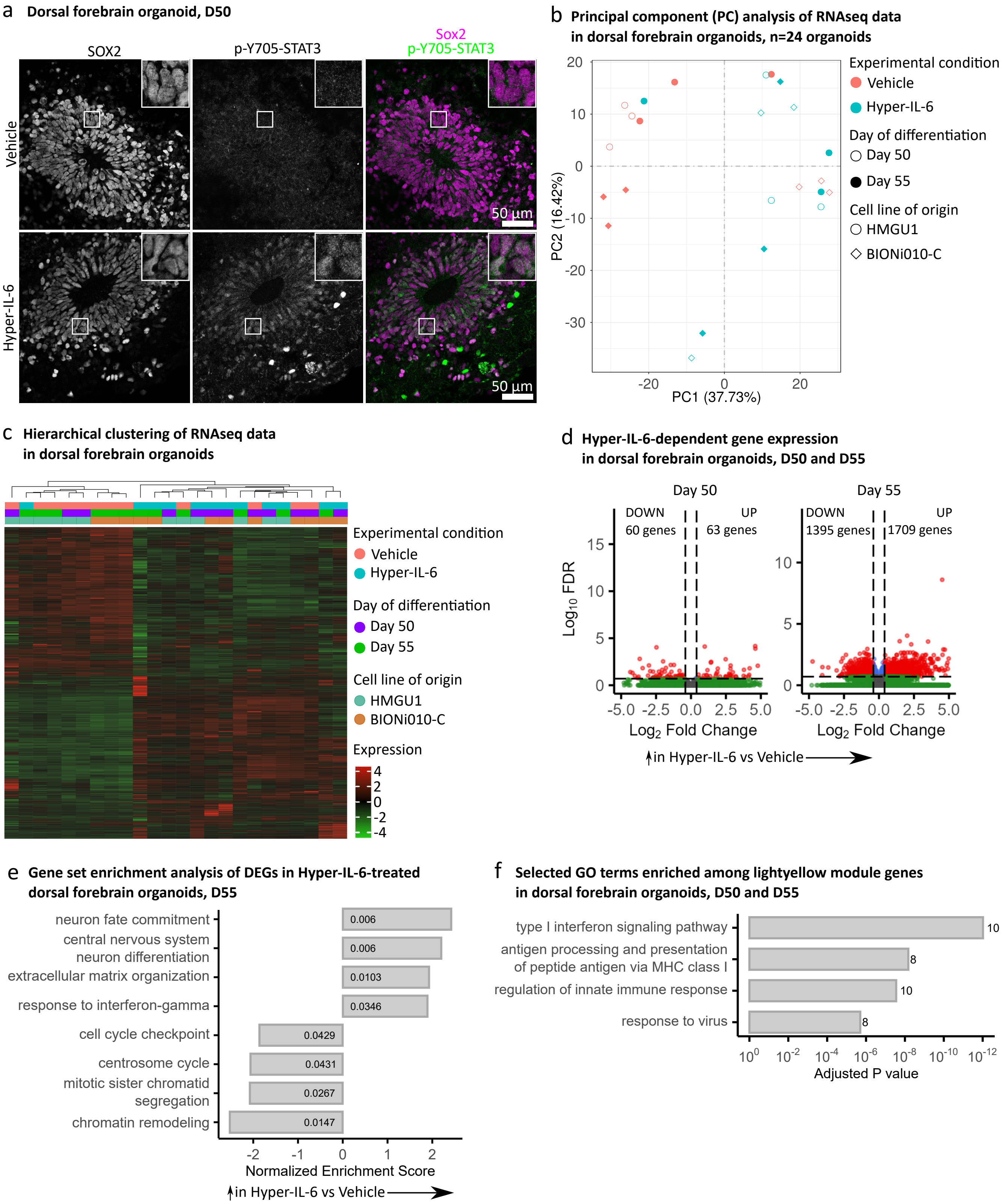

Supplement: Supplementary file 3 — Figure S2 [file 41380_2023_1997_MOESM3_ESM.pdf]
