# Supplementary material for: Human brain organoid model of maternal immune activation identifies radial glia cells as selectively vulnerable
Source: Mol Psychiatry. 2023 Mar 6;28(12):5077–89. doi: 10.1038/s41380-023-01997-1 (PMC9986664; doi:10.1038/s41380-023-01997-1)

Supplementary Figure 3

a Ventricular-like zones in dorsal forebrain organoids, D50

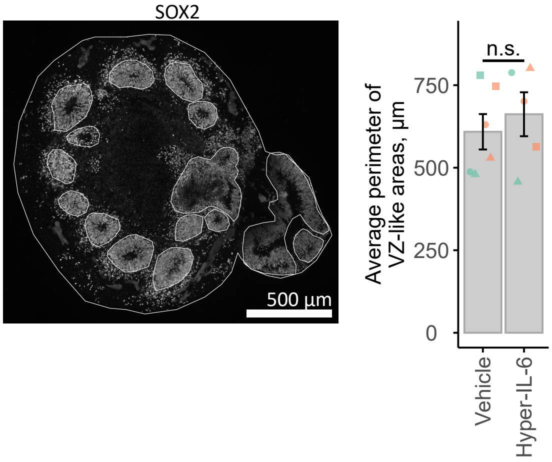

b Ventricular-like zones in dorsal forebrain organoids, D55

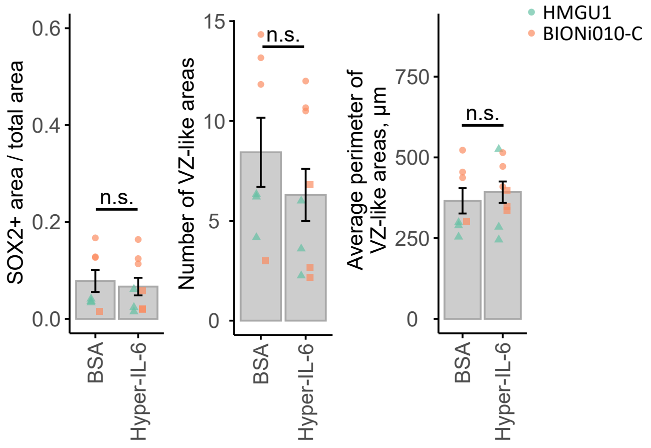

c vRGs in dorsal forebrain organoids, D55

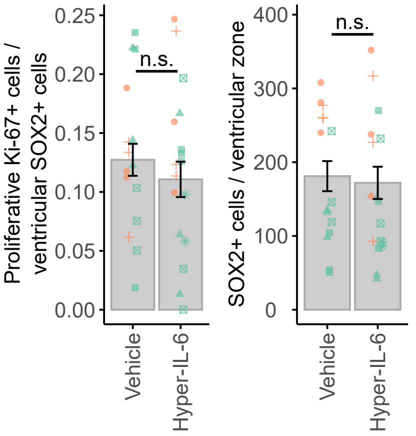

d Neurogenesis over 100 µm of ventricular surface in dorsal forebrain organoids, D50

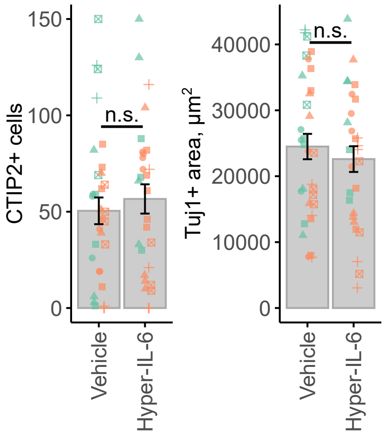

e Proportion of double CTIP2- and SATB2-positive neurons in dorsal forebrain organoids, D90

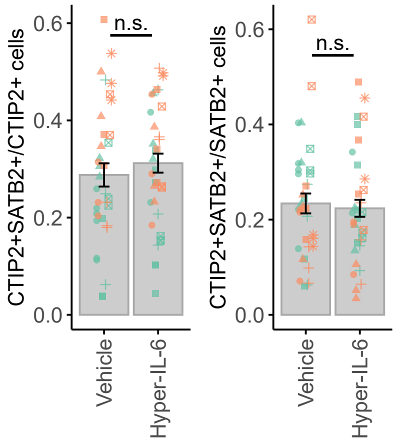

f Distribution of deep- and upper-layer neurons in dorsal forebrain organoids, D90

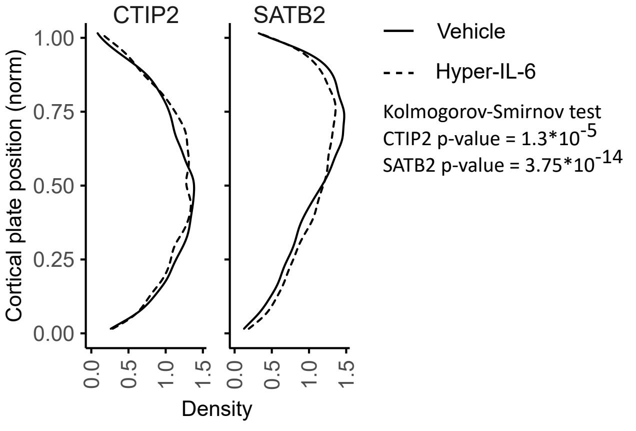

Supplement: Supplementary file 4 — Figure S3 [file 41380_2023_1997_MOESM4_ESM.pdf]
