# Supplementary material for: Human brain organoid model of maternal immune activation identifies radial glia cells as selectively vulnerable
Source: Mol Psychiatry. 2023 Mar 6;28(12):5077–89. doi: 10.1038/s41380-023-01997-1 (PMC9986664; doi:10.1038/s41380-023-01997-1)

Supplementary Figure 5

a Venn diagram of differentially downregulated genes in cycling vRGs and RGs from Kalish *et al., 2021*

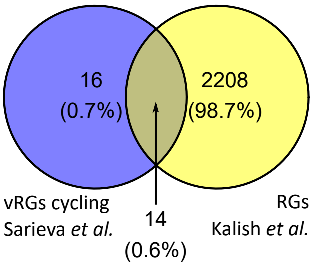

b Gene set overrepresentation analysis of 14 differentially downregulated genes overlapping in cycling vRGs and RGs from Kalish *et al., 2021*

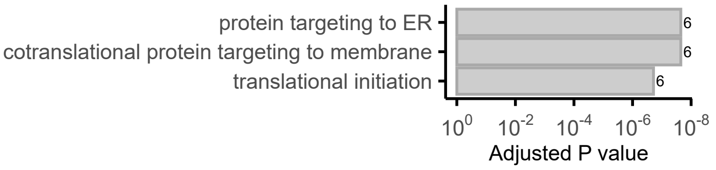

c Expression of gene modules related to ASD based on Satterstrom *et al., 2020*

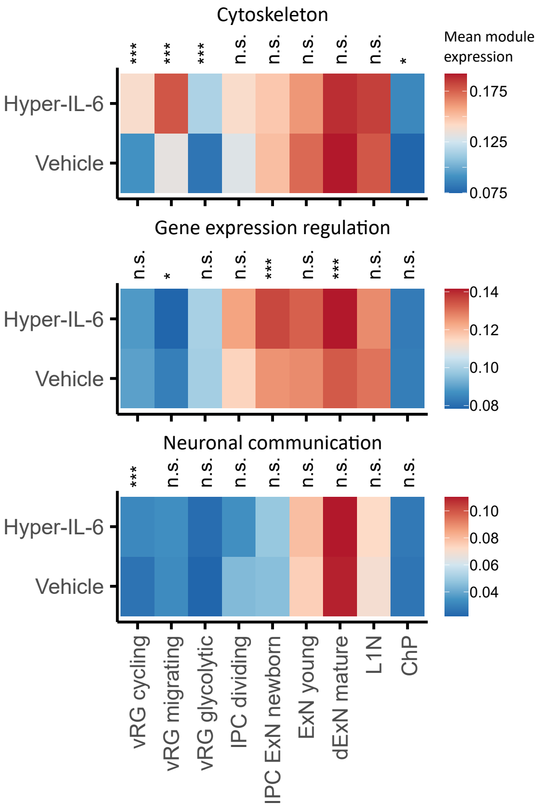

d Relevance of Hyper-IL-dependent gene expression changes to ASD

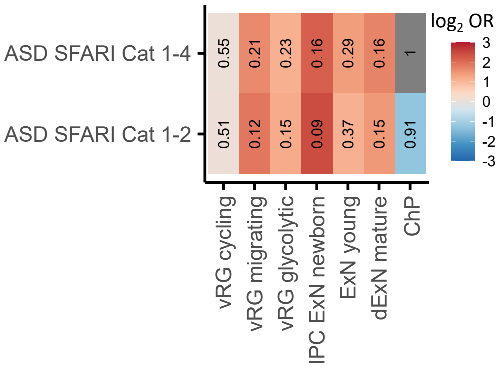

e Gene set overrepresentation analysis of the genes within STAT3 regulon in cycling vRGs

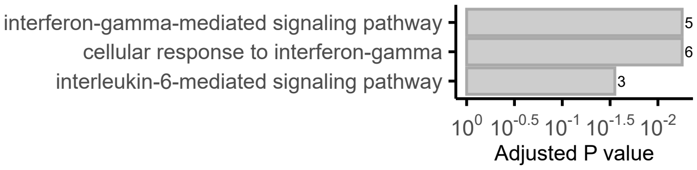

f Gene set overrepresentation analysis of the genes within NR2F1 regulon in cycling vRGs

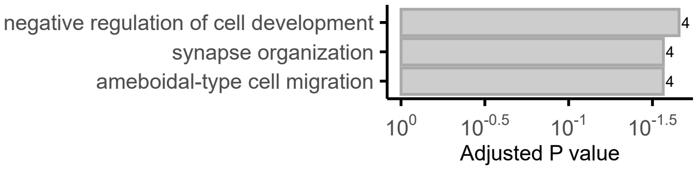

g Genes within NR2F1 regulon in cycling vRGs

Gene name	Protein name
ANK2	ankyrin 2
CLU	clusterin
ERBB4	erb-b2 receptor tyrosine kinase 4
GLIPR1	GLI pathogenesis related 1
GPC3	glypican 3
LRRN3	leucine rich repeat neuronal 3
NFIA	nuclear factor I A
NPPC	natriuretic peptide C
NR2F1	nuclear receptor subfamily 2 group F member 1
NR2F2	nuclear receptor subfamily 2 group F member 2
SEMA5A	semaphorin 5A
SPARCL1	SPARC like 1

h Dorsal forebrain organoid, D50

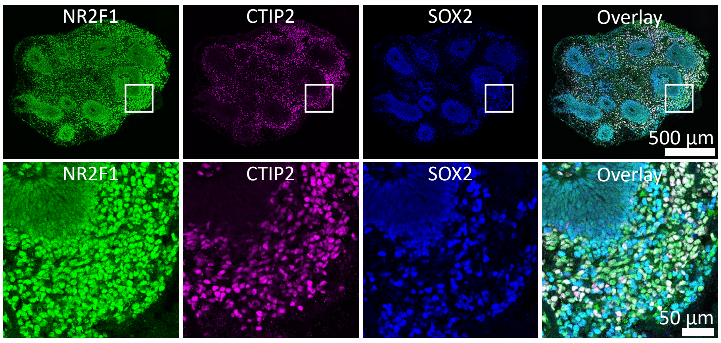

Supplement: Supplementary file 6 — Figure S5 [file 41380_2023_1997_MOESM6_ESM.pdf]
