# Supplementary material for: Human brain organoid model of maternal immune activation identifies radial glia cells as selectively vulnerable
Source: Mol Psychiatry. 2023 Mar 6;28(12):5077–89. doi: 10.1038/s41380-023-01997-1 (PMC9986664; doi:10.1038/s41380-023-01997-1)
Supplement: Supplementary file 7 — Supplementary Fig. legends [file 41380_2023_1997_MOESM7_ESM.pdf]

Supplementary Figure 1. Related to Figure 1. Characterization of IL-6 cascade in human neocortex and dorsal forebrain organoids. a, IL6ST (magenta) immunofluorescence of human PCW13 neocortex, shows protein expression in the VZ and SVZ. Nestin (green) is used as a marker of RG cells. VZ, ventricular zone; SVZ, subventricular zone; IZ, intermediate zone; CP, cortical plate; MZ, marginal zone. b, Confocal tilescan image across human neocortex at PCW12 immunostained for IL6ST (magenta) and PPP1R17 (green). c, p-Y705-STAT3 immunofluorescence in human neocortex at PCW13 shows co-localization with the marker of RGs, SOX2. d, At day 50 of differentiation, DFOs consist preferentially of SOX2-positive RGs, TBR2-positive IPCs and CTIP2-positive dExNs. e, Excitatory lineage of the human neocortical cells at PCW9-15 largely consists of the radial glia, intermediate progenitor cells, newborn and mature neurons in line with organoid cell type composition based on single-cell transcriptomes from Nowakowski *et al*, Science, 2017.

Supplementary Figure 2. Related to Figure 2. Quality control of RNAseq data. a, p-Y705-STAT3 immunofluorescence in DFOs at D50 shows co-localization with the marker of RGs, SOX2, upon Hyper-IL-6 but not Vehicle treatment. b, Principal component analysis of RNAseq data from n=24 organoids from 2 iPSCs cell lines (BIONi010-C and HMGU1), two time points over organoids differentiation (D50 and D55) and two experimental conditions (Vehicle and Hyper-IL-6). c, Hierarchical clustering of RNAseq data showing clustering of samples on the basis of experimental condition, day of differentiation and iPSC line. Clustering was performed based on all expressed genes. d, Volcano plot of Hyper-IL-6-dependent gene expression in DFOs at days 50 and 55 of differentiation treated for 5 and 10 days, respectively. Red dots indicate statistical significance (FDR < 0.2, absolute log2 Fold Change > 0.4). Positive log2 Fold Change indicates higher gene expression in Hyper-IL-6-treated relative to Vehicle-treated DFOs. Data from n=6 organoids per condition per day of differentiation (two cell lines). e, GSEA of DEGs (FDR < 0.2, log2 Fold Change > 0.4) between Hyper-IL-6 and Vehicle-treated organoids at day 55 of differentiation. The x axis displays normalized enrichment score. Numbers inside the bars represent adjusted p-values for differential enrichment. f, ORA of genes constituting light-yellow WGCNA module. The x axis displays the adjusted p-value. Numbers next to the bars represent the number of DEGs belonging to the GO term. Data from n=12 organoids per condition (two cell lines, day 50 and 55 analyzed together).

Supplementary Figure 3. Related to Figure 3. Hyper-IL-6 treatment does not result in changes in cell type composition in DFOs by day 55 of differentiation. a, Hyper-IL-6 treatment does not lead to changes in the average perimeter of SOX2-positive areas. Four sections of each DFO were analyzed. Representative tilescan image displays an exemplary analysis workflow. White lines outline the organoid and SOX2-positive areas within it. In the plot, each dot represents the mean value of four sections in one single organoid. Color represents cell line of origin, HMGU1 (orange), BIONi010-C (green). Vehicle (n = 6) and Hyper-IL-6 (n = 5) DFOs from one batch per iPSC line. b, Hyper-IL-6 treatment does not lead to changes in the average perimeter of SOX2-positive areas, their number and area at day 55 of organoids differentiation. Six sections of each DFO were analyzed. Each dot represents the mean value of six sections in one single organoid. Color represents cell line of origin, HMGU1 (green), BIONi010-C (orange). Vehicle (n = 7) and Hyper-IL-6 (n = 9) DFOs both iPSC lines. c, Hyper-IL-6 treatment does not lead to changes in proportion of Ki-67-positive proliferative cells over SOX2-positive vRGs on day 55 of differentiation. Hyper-IL-6 does not lead to an increase in SOX2-positive vRGs on day 55 of differentiation. Each dot represents individual VZ-like region, 2-3 regions imaged. Color represents cell line of origin and batch of differentiation, HMGU1 (green and violet), BIONi010-C (green). Shape corresponds to individual DFO. Vehicle (n = 7) and Hyper-IL-6 (n = 8) DFOs from both iPSC lines. d, Hyper-IL-6 treatment does not lead to changes in CTIP2-positive dExNs as well as in the Tuj1-positive area at day 50 of differentiation. Each dot represents individual region of interest with VZ-like zone, 2-5 regions imaged per organoid. Color represents cell line of origin, HMGU1 (green), BIONi010-C (orange). Shape corresponds to individual DFO. Vehicle (n = 10) and Hyper-IL-6 (n = 8) DFOs from one batch per iPSC line. e, Hyper-IL-6 treatment does not lead to cell fate misspecification in CTIP2-positive dExNs and SATB2-positive uExNs at day 90 of differentiation shown as proportion of double-positive cell per total number of CTIP2-/SATB2-positive cells. Each dot represents individual region-of-interest representing a putative CP-like region, 2-3 regions per DFO imaged. Color represents cell line of origin, HMGU1 (green), BIONi010-C (orange). Shape corresponds to individual DFO. Vehicle (n = 12) and Hyper-IL-6 (n = 14) DFOs from three batches. The

same samples as in Figure 3f are shown. f, Hyper-IL-6 treatment leads to incorrect laminar positioning of both CTIP2-positive dExNs and SATB2-positive uExNs at day 90 of differentiation. The position of each cell was normalized with respect to the cortical structure from the surface of the organoid until the end of dense CTIP2-positive area. The curves represent the normalized abundance of cells along the putative CP. Same samples as in Figure 3f are shown. Kolmogorov-Smirnov test. In panels a-e, bars represent mean, error bars represent ± SEM. Comparisons were analyzed using Aligned Rank Transform (ART) ANOVA: n.s., non-significant p-value > 0.05.

Supplementary Figure 4. Related to Figure 4. Quality control of scRNAseq and cell type composition analysis. a, Total number of genes per sample and log10 of genes per UMI per sample in the single cell sequencing dataset. b, Heatmap of physiological feature assessment across clusters. Distribution of the cells in each cluster across cell cycle phases (top), expression of module signature genes for GO terms related to Neurogenesis (GO:0022008, middle) and Hypoxia (GO:0001666, bottom) across cell clusters. Colors in the lower bars represent cell types from Figure 4b. c, Automated assignment of cell cluster identity to the dataset consisting of the human neocortical cells (from *Zhong et al.*, Nature, 2018) and dorsal forebrain organoid cells (from *Velasco et al.*, Nature, 2019) with cell type labels from *Tanaka et al.*, Cell Reports, 2020. Cell frequency is presented column-wise. Cell type labels by Tanaka and colleagues: NEC, neuroepithelial cell; OL, oligodendrocyte; GPC, glia progenitor cell; PGC, proteoglycan-expressing cell; UPRC, unfolded protein response-related cell; BRC, BMP-related cell; AS, astrocyte; CN, cortical excitatory neuron; Inter, interneuron. d, Heatmap of similarity metric of VoxHunt algorithm comparing organoid clusters with human brain RNAseq data from BrainSpan using brain regional markers obtained from Mouse Brain Atlas at E13. Colors in the left bar represent cell types from Figure 4b. e, UMAP with color representing cell line of origin, HMGU1 (green), BIONi010-C (orange). f, Permutation test on cell type composition of Hyper-IL-6-treated organoids. Differentially abundant cell types are represented in pink. FDR less than 0.05 and absolute log2 fold change more than 0.58 were considered differentially abundant. In all panels, data from n = 4 organoids from two cell lines (HMGU1 and BIONi010-C) and two experimental conditions (Vehicle and Hyper-IL-6).

Supplementary Figure 5. Related to Figure 5. Single-cell DGE and transcriptional networks changes upon Hyper-IL-6 treatment. a, Venn diagram showing the intersection of differentially downregulated genes from cycling vRG metacluster and RG cells from Kalish and colleagues. b, ORA of overlapping differentially downregulated genes from a. The x axis displays the adjusted p-value. Numbers next to the bars represent the number of DEGs belonging to the GO term. c, Heatmap representing mean module expression of ASD-relevant gene groups by treatment condition across cell clusters from Figure 5a. Comparisons were analyzed using t-test, Bonferroni adjusted p-values: n.s., non-significant p-value > 0.05; *, p-value < 0.05; ***, p-value < 0.001. d, Enrichment of ASD risk genes and among DEGs upon Hyper-IL-6 treatment. Log2 odds ratio (OR) is represented by color and adjusted p-values are indicated inside the tiles. Fisher's exact test; p-values corrected for multiple comparisons by Benjamini-Hochberg method. ASD SFARI Cat 1-4 corresponds to ASD SFARI risk genes from categories 1-4 from SFARI release on 31.10.2019 (old classification); ASD SFARI Cat 1-2 corresponds to ASD SFARI risk genes from categories 1-2 from SFARI release on 20.07.2022 (new classification). e,f, ORA of genes belonging to STAT3 (e) and NR2F1_extended (f) regulons as assessed by SCENIC. The x axis displays the adjusted p-value. Numbers next to the bars represent the number of the genes belonging to the GO term. g, Genes belonging to the NR2F1_extended regulon as assessed by SCENIC. h, At day 50 of differentiation, dorsal forebrain organoids express NR2F1 in both SOX2-positive RGs and CTIP2-positive dExNs.
